# Intrathecal injection of 3D-mesenchymal stem cells attenuates disseminated neuroinflammation and improves cognitive function in controlled cortical impact rats

**DOI:** 10.3389/fnins.2026.1895538

**Published:** 2026-07-28

**Authors:** Rui Xu, Wenwen Zhang, Zizi He, Yunyun Huo, Yaojiong Wu, Yankai Dong, Chenglin Tian, Ye Ran

**Affiliations:** 1 Department of Neurology, The First Medical Center of Chinese PLA General Hospital, Beijing, China; 2 Huadong Medicine Co., Ltd., Hangzhou, China; 3 State Key Laboratory of Chemical Oncogenomics, and Institute of Biopharmaceutical and Health Engineering (iBHE), Tsinghua Shenzhen International Graduate School, Tsinghua University, Shenzhen, China; 4 Institute of Cell and Gene Technology, Shenzhen University of Advanced Technology, Shenzhen, China; 5 Tsingyuan Biotech (Guangdong) Co., Ltd., Heyuan, China

**Keywords:** cognitive function, controlled cortical impact, mesenchymal stem cells, neuroinflammation, traumatic brain injury

## Abstract

**Background:**

Persistent cognitive impairment following traumatic brain injury (TBI) represents a significant unmet clinical need. Neuroinflammation has been implicated as one of the pathological processes that may contribute to this outcome. Given the limitations of current therapies, this study explored the therapeutic potential of three-dimensional cultured mesenchymal stem cells (3D-MSCs) for TBI, with a focus on long-term cognitive outcome and their immunomodulatory properties.

**Methods:**

A controlled cortical impact (CCI) model was established in male Sprague-Dawley rats. Twenty-four hours post-injury, rats received an intrathecal injection of 1x10^6^ 3D-MSCs or vehicle. Cell migration, apoptosis, lesion volume, sensorimotor and cognitive functions, and neuroinflammation were subsequently evaluated.

**Results:**

Intrathecally administered 3D-MSCs exhibited significant homing to the perilesional region within 24 h. Subsequently, the treatment reduced perilesional apoptosis by 59.0% at 48 h and lesion volume by 28.8% at 7 days post-injury (dpi). 3D-MSCs significantly enhanced sensorimotor recovery from day 3 and improved spatial learning and memory at 30 dpi. Importantly, it also attenuated CCI-induced widespread Iba1-associated neuroinflammatory responses across multiple brain regions at 7 dpi.

**Conclusion:**

Focal TBI can induce disseminated neuroinflammation. A single intrathecal injection of 3D-MSCs within 24 h after TBI enables rapid migration to the injury site and exerts early neuroprotective effects. In addition, 3D-MSC administration improved long-term cognitive function and attenuated disseminated neuroinflammatory responses, suggesting that immunomodulation may be one of the factors contributing to its therapeutic benefit.

## 1 Introduction

Annually, over 50 million individuals worldwide are affected by TBI, which is associated with high rates of mortality and disability (GBD 2016 Traumatic Brain Injury and Spinal Cord Injury Collaborators, 2022). Increases in life expectancy and motor vehicle use have contributed to the rising incidence and prevalence of TBI (Dams-O’Connor et al., 2023). Current management primarily focuses on supportive measures to reduce acute mortality by stabilizing hemodynamics and controlling intracranial pressure (Robba et al., 2025). Nonetheless, TBI frequently leads to persistent cognitive deficits (Schneider et al., 2024), profoundly affecting patients’ quality of life and social functioning. However, effective management of post-TBI cognitive impairment remains a major challenge.

A growing body of clinical evidence indicates that cognitive impairment following TBI commonly arises from disruption of cognition-related neural networks (Patil et al., 2025; Sanker et al., 2025; Xu et al., 2026). However, the pathophysiological processes underpinning damage to these critical brain connectomes remain poorly understood. Neuroinflammation, particularly diffuse neuroinflammatory responses extending beyond the primary injury site, may represent a pivotal driving factor. Increasing clinical research suggests that neuroinflammatory changes may be widely distributed within key hub regions of cognition-related networks (Shi et al., 2019; De Picker et al., 2023). As the major immune cells within the central nervous system, microglia can disrupt white matter connections between brain regions by affecting myelin and axonal integrity and can also directly regulate synaptic plasticity and neuronal activity (Hou et al., 2024; Feichtenbiner et al., 2025). Preclinical evidence indicates that post-traumatic neuroinflammatory responses involving microglia are closely associated with the severity of cognitive deficits (Saba et al., 2021). This suggests that widespread neuroinflammatory responses within critical nodes of cognitive networks may ultimately disrupt circuit function by impairing the integrity of both network connectivity (white matter) and information-processing units (gray matter synapses). However, most previous basic studies have focused on post-TBI neuroinflammation within the perilesional area or the hippocampus, with inadequate attention given to the more clinically relevant diffuse, network-level inflammatory response. Therefore, further research is required to deepen our understanding of and intervention in this diffuse neuroinflammatory response. This may offer novel therapeutic strategies and targets for restoring impaired neural network function and improving cognitive outcomes following TBI.

Pharmacological or genetic elimination of microglia has been demonstrated to suppress early neuroinflammation and improve outcomes following TBI (Delcy et al., 2026). However, the inflammatory response is a “double-edged sword” (Jia et al., 2025), as it also plays a crucial role in clearing necrotic tissue and cells during injury repair. Therefore, precise modulation, rather than complete elimination, of the diffuse inflammatory response following injury may represent a potential therapeutic strategy for TBI. Beyond their characterization as mesoderm-derived multipotent stem cells, MSCs are primarily distinguished by their potent immunomodulatory and paracrine functions, which underpin their significant therapeutic potential in complex inflammatory environments (Wang et al., 2025). Consequently, they have gradually emerged as a highly promising therapeutic approach for TBI (Kabatas et al., 2024). Compared with conventional adherent-cultured MSCs, 3D-MSCs exhibit superior engraftment and homing capacity, immunomodulatory function, and amplified paracrine effects (Bartosh et al., 2010; Chen et al., 2025). In this study, “3D-MSCs” refers to MSCs preconditioned by 3D spheroid culture and administered as a dissociated single-cell suspension rather than as intact spheroids. In addition, our previous research demonstrated that intrathecal administration of 3D spheroid culture-preconditioned MSCs results in fewer complications, such as obstructive hydrocephalus and ventricular dilation, than 2D-MSCs, indicating a superior safety profile (Ran et al., 2022). Nevertheless, to our knowledge, the therapeutic potential of 3D-MSCs for TBI has not yet been investigated.

**FIGURE 1:**
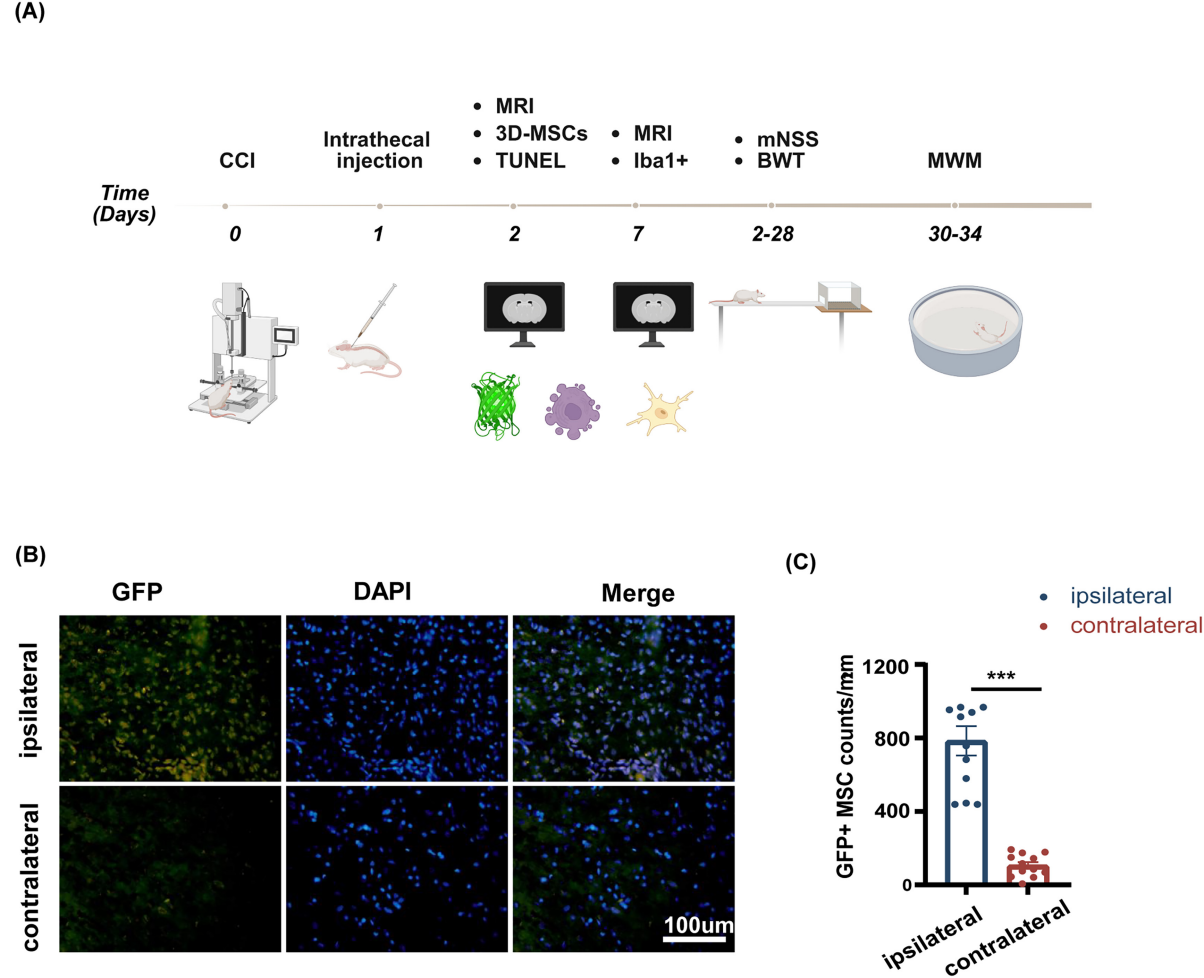
Intrathecally administered 3D-MSCs rapidly home to the injured hemisphere after CCI. **(A)** Experimental design. Rats received an intrathecal injection of 3D-MSCs or an equal volume of ACSF at 24 h after CCI. Lesion volume was evaluated by T2-weighted MRI at 2 and 7 dpi. GFP-labeled cells and TUNEL staining were assessed at 2 dpi, and Iba1 immunofluorescence was quantified at 7 dpi. Sensorimotor function was assessed by mNSS and BWT from 1 to 28 dpi, and cognitive function was assessed by MWM at 30–34 dpi. **(B)** Representative fluorescence images showing GFP-labeled 3D-MSCs in the ipsilateral and contralateral hemispheres. **(C)** Quantification of GFP-positive cells showing significantly greater accumulation in the ipsilateral hemisphere than in the contralateral hemisphere. Data are shown as mean ± SD; *n* = 4 per group. ***p < 0.001. ACSF, artificial cerebrospinal fluid; BWT, beam-walking test; CCI, controlled cortical impact; GFP, green fluorescent protein; Iba1, ionized calcium-binding adapter molecule 1; mNSS, modified neurological severity score; MRI, magnetic resonance imaging; MSCs, mesenchymal stem cells; MWM, Morris water maze; TUNEL, terminal deoxynucleotidyl transferase-mediated nick-end labeling.

In this study, we aimed to characterize neuroinflammatory responses and neurological outcomes following focal TBI and to evaluate the therapeutic potential of 3D-MSCs (schematic overview of the overall research design shown in Figure 1A). We hypothesized that focal TBI induces a diffuse neuroinflammatory response in brain regions related to cognitive function, which may be associated with subsequent cognitive impairment. We further postulated that a single intrathecal injection of 3D-MSCs might improve cognitive outcomes, potentially in part through modulation of this widespread inflammatory response.

## 2 Materials and methods

### 2.1 Animals

Animal experiments were conducted using male Sprague-Dawley rats (250–280 g) obtained from Sipeifu Biotechnology Co., Ltd., (Beijing, China). Animals were housed under standard conditions, with temperature maintained at 22 ± 2°C, humidity at 50 ± 10%, and a 12/12-h light/dark cycle, with free access to food and water. All experimental procedures were approved by the Medical Ethics Committee of Chinese PLA General Hospital (ethics approval number: 2024-512-01) and were conducted in compliance with the Guide for the Care and Use of Laboratory Animals, including protocols for humane endpoints and euthanasia to minimize suffering.

### 2.2 Cell culture

MSCs were provided by the State Key Laboratory of Chemical Oncogenomics (Tsinghua University, Shenzhen International Graduate School). Briefly, MSCs were obtained with written informed consent from donors and isolated from human placenta according to previously established protocols (Guo et al., 2014). All procedures were approved by the ethics committees of Peking University Shenzhen Hospital and Tsinghua University, China. For 2D expansion, cells were cultured in DMEM (Corning) supplemented with 10% FBS (Biological Industries) under standard conditions (37°C, 5% CO_2_) and passaged at 80% confluence by trypsinization. For 3D spheroid generation, passage 5–7 MSCs were suspended in 35-μL drops of DMEM containing 10% FBS (20,000 cells/drop), incubated for 36 h, and then transferred to suspension culture for 24 h. The spheroid diameter before dissociation was approximately 200–300 μm. For tracking, MSCs were transduced with lentiviral GFP vectors (Cyagen) according to the manufacturer’s instructions. Finally, the spheroids were enzymatically dissociated into single-cell suspensions using 0.25% trypsin/EDTA with intermittent pipetting, followed by cryopreservation for subsequent experiments. After thawing, the cell viability was 96.28%, and the cell recovery rate after filtration was 98.34%. MSC identity was confirmed by flow cytometric analysis of MSC-specific surface markers. Therefore, the injected product consisted of 3D culture-preconditioned MSCs rather than intact spheroids. Detailed quality-control data are provided in Supplementary Text 1.

### 2.3 Controlled cortical impact model

The CCI injury model was established using a precision cortical impactor (68099II, RWD Inc., China) with a rounded steel impactor probe (5 mm diameter) in accordance with standardized protocols (Dean et al., 2017). Rats were anesthetized with isoflurane (5% for induction, 1–2% for maintenance). Body temperature was maintained at 37.0 ± 0.5 °C during the procedure and recovery period. Following a ∼2-cm midline scalp incision to expose the cranial surface, a ∼6-mm circular craniotomy was performed centered at 3.0 mm posterior and 3.0 mm right lateral to bregma. Cortical impact was delivered perpendicularly to the dura at a velocity of 4.5 m/s and a depth of 2.5 mm with a dwell time of 1.0 s, resulting in moderate-to-severe CCI, as previously reported (Dean et al., 2017). The bone flap was repositioned and fixed with dental cement. Sham controls underwent identical procedures except for cortical impact.

### 2.4 Intrathecal transplantation of MSCs

In the CCI cohort, intrathecal MSC delivery (1 × 106 cells) was performed 24 h after injury. For transplantation, cryopreserved 3D-MSCs were rapidly thawed in a 37°C water bath, washed in 10 mL sterile phosphate-buffered saline (PBS), and resuspended in artificial cerebrospinal fluid (ACSF). The cell density was adjusted to 1 × 107 cells/mL. Following filtration through a 40-μm strainer to ensure a single-cell suspension, 100 μL of the suspension (1 × 106 cells) was infused into the cisterna magna over 5 min using a glass capillary connected to a 1-mL syringe via polyethylene tubing.

### 2.5 Early neurological function assessment

Early neurological function was assessed using the modified Neurological Severity Score (mNSS) and beam-walking test (BWT), standardized tools for quantifying motor and sensory deficits in rodent models. Before surgery, all rats underwent daily mNSS and BWT assessments for five consecutive days to acclimate them to the testing procedures and establish baseline performance. Postoperative evaluations were conducted on days 1, 2, 3, 5, 7, 9, 11, 13, 15, 17, 19, 21, and 28 by two independent observers blinded to group allocation. The mNSS evaluates motor function, sensory responses, and reflexes in rats. Scores range from 0 (intact function) to 18 (maximal deficit), with higher values indicating more severe impairment. Motor coordination was quantified using the BWT. Rats traversed a narrow beam (122 × 2.5 cm) under controlled aversive stimulation (60-W light and 85-dB white noise). The key metrics included traversal latency (cutoff: 60 s) and foot-fault frequency (limb slips or grasps below the beam surface). Three trials were performed each day, and the data were averaged across trials for statistical analysis.

### 2.6 Morris water maze

The Morris water maze (MWM) was conducted on days 30–34 after CCI to assess spatial learning and memory function using a circular water maze pool (diameter, 160 cm; height, 80 cm) filled with water (25 ± 2°C) rendered opaque with titanium dioxide. On postoperative days 30–33, rats underwent a place-navigation test (4 trials/day) with a transparent platform (diameter, 12 cm) submerged 1–2 cm below the surface in a fixed quadrant. For each trial, rats were released facing the wall from one of four randomized entry points (NE, NW, SE, and SW quadrants), and escape latency (time to locate the platform within 120 s) was recorded. Daily performance was calculated as the mean latency across four trials. On day 34, a probe trial was conducted in which the platform was removed and rats were released from a randomized quadrant to swim freely for 120 s. Movement trajectories and time spent in the target quadrant were analyzed using an automated video-tracking system (SuperMaze v3.0, Shanghai Xinruan, China).

### 2.7 Magnetic resonance imaging

On postoperative days 2 and 7, lesion location and volume were evaluated using a 7.0-T high-field small-animal magnetic resonance imaging (MRI) system (Bruker BioSpin, Ettlingen, Germany) with a commercial rat coil (diameter, 38 mm). T2-weighted imaging (T2WI) was performed with the following parameters: repetition time (TR), 2500 ms; echo time (TE), 33 ms; and slice thickness, 0.6 mm. Rats were anesthetized by inhalation of 4–5% isoflurane for induction and maintained at 2% isoflurane. Animals were secured in a stereotaxic holder with continuous gas delivery through a nose cone. Hyperintense lesions on T2WI were manually delineated by an observer blinded to the experimental groups using ITK-SNAP software (v4.0.1, National Institutes of Health, MD, USA). Lesion volume (mmł) was automatically calculated based on the annotated regions of interest (ROIs) according to standard protocols.

### 2.8 Tissue harvesting and preservation

Following anesthesia, rats underwent transcardial perfusion with PBS and 4% paraformaldehyde (PFA) at predetermined postinjury intervals. Harvested brains were postfixed in 4% PFA for 24 h, equilibrated in 30% sucrose until osmotic saturation (defined as tissue sinking), and cryopreserved at -80°C for further analysis. For spatial mapping of 3D-MSCs and apoptosis detection, coronal sections (10 μm thick) spanning 2.52–3.48 mm posterior to bregma (perilesional zone) were stored in PBS. For neuroinflammatory analysis, tissues from 2.16–1.20 mm anterior and 2.64–3.36 mm posterior to bregma were sectioned at 30 μm thickness. Sections were sampled at 1:4 intervals and stored in PBS for free-floating immunofluorescence staining.

### 2.9 General histological procedures

Unless otherwise specified, all brain sections were subjected to three 5-min washes in PBS on an orbital shaker (40 rpm) at room temperature. Following the specific staining protocols, sections were counterstained and mounted with DAPI-containing mounting medium (or Fluoroshield). Fluorescent images were acquired using an upright fluorescence microscope (Nikon, Japan) by an observer blinded to the experimental groups.

### 2.10 Temporal and spatial analysis of transplanted 3D-MSCs

Brain tissues were harvested 24 h after transplantation to analyze the distribution of GFP-labeled 3D-MSCs. Coronal sections were processed according to the general histological procedures described above. GFP^+^ cells were quantified within the perilesional zone (defined as a 1-mm perimeter surrounding the necrotic tissue) and the contralateral homotopic cortex.

### 2.11 TUNEL staining

To detect apoptosis, 10-μm sections were permeabilized with 0.5% Triton X-100 in PBS for 5 min. After PBS washes, the TUNEL reaction mixture (C1090, Beyotime, China) was applied for 60 min at room temperature under light-restricted conditions. After final washes and DAPI mounting (see General histological procedures), TUNEL^+^ cells within the perilesional zone were quantified in three randomly selected fields per section (*n* = 4 per group).

### 2.12 Immunofluorescence staining

Brain sections were incubated overnight at 4°C with rabbit anti-Iba1 primary antibody (1:200, ab289370, Abcam). After washes, sections were incubated for 2 h at room temperature in the dark with Alexa Fluor 594-conjugated secondary antibody (1:500, ab150084, Abcam). Following the general washing and mounting procedures, Iba1-positive signals were quantified across nine brain regions in both the ipsilateral and contralateral hemispheres using ImageJ. Positive signals were defined as fluorescence intensity exceeding three times the mean background signal.

### 2.13 Statistical analyses

Data were analyzed using SPSS 18.0 statistical software (SPSS Inc., Chicago, IL, USA). Continuous data are presented as mean ± SD. Normality was assessed using the Shapiro–Wilk test (*p* > 0.05), and homogeneity of variance was assessed using Levene’s test (*p* > 0.10). Normally distributed data were analyzed using one-way ANOVA followed by Tukey’s *post hoc* test, whereas non-normally distributed data were analyzed using the Kruskal–Wallis test followed by Dunn’s multiple-comparison test. For histological analyses, the animal was considered the biological unit of analysis; multiple sections from the same animal were treated as technical replicates and averaged prior to group-level statistical testing. For the analyses shown in Figure 5, the nine brain regions were predefined anatomical regions of interest and were analyzed as planned, region-specific comparisons. For longitudinal behavioral measurements (mNSS, BWT, and the MWM navigation test), repeated-measures ANOVA was applied, followed by Tukey’s *post hoc* test for multiple comparisons. MRI data were additionally analyzed using paired or unpaired *t*-tests, as appropriate. Details are provided in the Supplementary Text 2. Statistical significance was set at *p* < 0.05 for all tests.

## 3 Result

### 3.1 Intrathecal 3D-MSCs rapidly home to the perilesional region after CCI

To determine whether intrathecally delivered 3D-MSCs could migrate toward the site of injury, GFP-labeled cells were examined at 2 dpi, corresponding to 24 h after intrathecal administration. Fluorescence imaging showed clear accumulation of GFP-positive cells in the injured hemisphere, particularly in the perilesional region, whereas markedly fewer cells were observed in the contralateral hemisphere (Figure 1B).

Quantitative analysis confirmed significantly greater numbers of GFP-positive cells in the ipsilateral hemisphere than in the contralateral hemisphere (Figure 1C, *P* < 0.001). These findings indicate that intrathecally administered 3D-MSCs can rapidly home to the injured hemisphere after CCI.

### 3.2 3 D-MSCs reduce perilesional apoptosis and lesion volume after CCI

**FIGURE 2:**
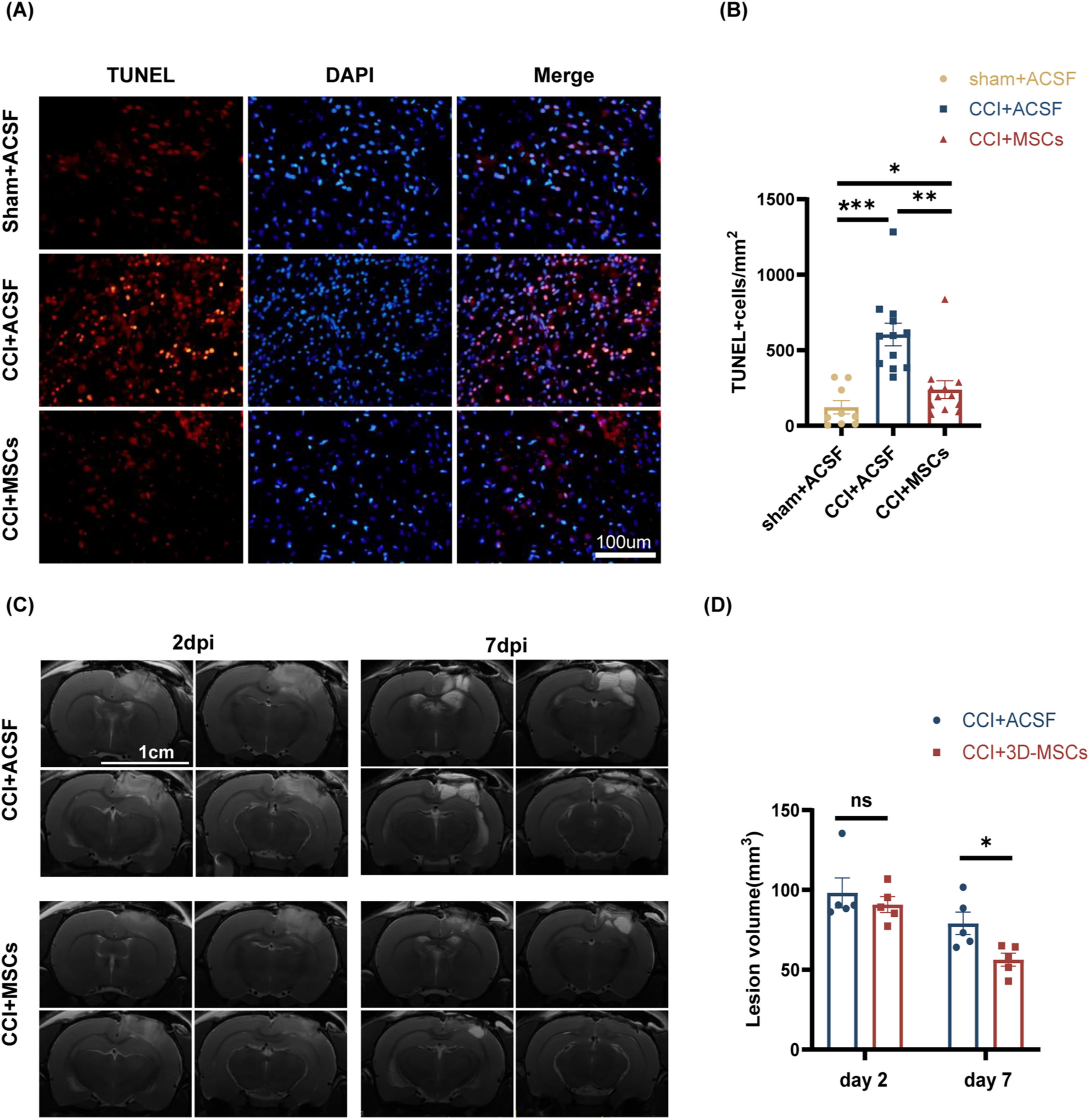
3D-MSCs reduce perilesional apoptosis and lesion volume after CCI. **(A)** Representative images of TUNEL staining in the perilesional region at 2 dpi. **(B)** Quantification of TUNEL-positive cells showing reduced apoptosis in the CCI + 3D-MSCs group compared with the CCI + ACSF group. **(C)** Representative T2-weighted coronal MRI images from rats subjected to CCI at 2 dpi and 7 dpi. The right side of each image corresponds to the rat’s right side, and the left side corresponds to the rat’s left side. Scale bars are shown. **(D)** Quantification of lesion volume showing a significant reduction in the CCI + 3D-MSCs group at 7 dpi. Data are shown as mean ± SD; *n* = 4 per group in panel **(B)** and *n* = 5 per group in panel **(D)**. *p < 0.05, **p < 0.01, ***p < 0.001; ns, not significant. ACSF, artificial cerebrospinal fluid; CCI, controlled cortical impact; dpi, days post-injury; MRI, magnetic resonance imaging; MSCs, mesenchymal stem cells; TUNEL, terminal deoxynucleotidyl transferase-mediated nick-end labeling.

To assess early neuroprotective effects, apoptosis in the perilesional region was examined by TUNEL staining at 2 dpi. Representative images are shown in Figure 2A. Quantitative analysis demonstrated that the number of TUNEL-positive cells was significantly lower in the CCI + 3D-MSCs group than in the CCI + ACSF group (Figure 2B), corresponding to a 59.0% reduction in perilesional apoptosis.

Lesion volume was evaluated by T2-weighted MRI at 2 and 7 dpi (Figure 2C). At 2 dpi, lesion volume did not differ significantly between the CCI + ACSF and CCI + 3D-MSCs groups. However, by 7 dpi, the CCI + 3D-MSCs group showed a significant reduction in lesion volume compared with the CCI + ACSF group (Figure 2D), corresponding to a 28.8% decrease. Additional within-group analysis showed that lesion volume significantly decreased from day 2 to day 7 in the 3D-MSCs group, but not in the ACSF group. Consistently, the lesion volume reduction rate was greater in the 3D-MSCs group than in the ACSF group (Supplementary Text 2). Collectively, these findings suggest that 3D-MSCs transplantation at 24 h post-CCI is associated with reduced perilesional apoptosis at 48 h and improved structural preservation during the subsequent post-injury period.

### 3.3 3 D-MSCs improve sensorimotor recovery after CCI

Neurological function was evaluated longitudinally using the mNSS and BWT from 1 to 28 dpi. Rats subjected to CCI exhibited marked neurological deficits compared with sham-operated animals. However, animals treated with 3D-MSCs showed significantly improved recovery over time.

**FIGURE 3:**
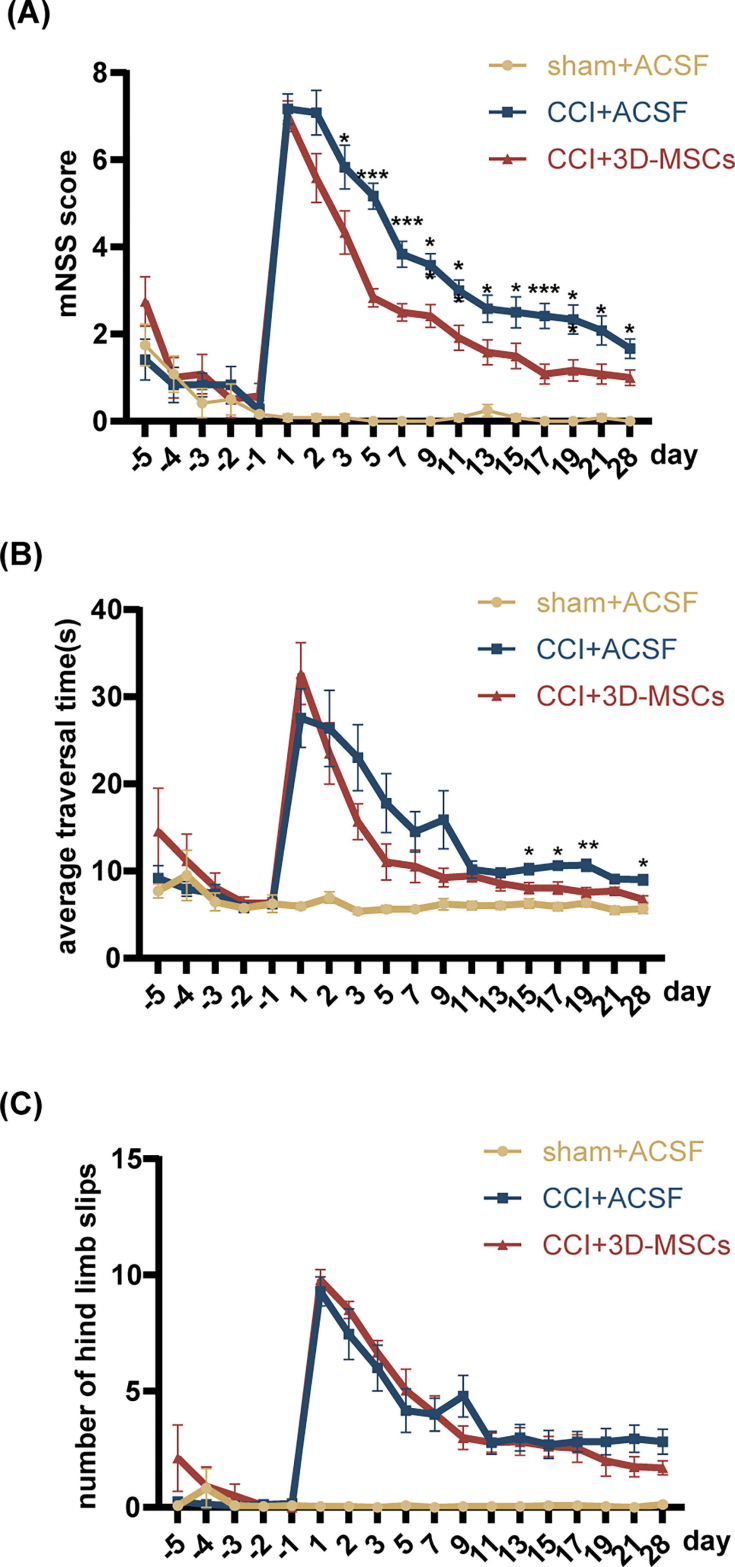
3D-MSCs improve sensorimotor recovery after CCI. Neurological function was evaluated by mNSS and BWT from 1 to 28 dpi. **(A)** Neurological function was evaluated by mNSS and BWT from 1 to 28 dpi. **(B)** Mean traversal time in the BWT. Rats in the CCI + 3D-MSCs group showed faster recovery than those in the CCI + ACSF group, with significant differences at later time points. **(C)** Hindlimb slip counts in the BWT. No significant differences were detected between the two CCI groups. Data are shown as mean ± SD; *n* = 12 per group. *p < 0.05, **p < 0.01, ***p < 0.001 versus CCI + ACSF. ACSF, artificial cerebrospinal fluid; BWT, beam-walking test; CCI, controlled cortical impact; mNSS, modified neurological severity score; MSCs, mesenchymal stem cells.

As shown in Figure 3A, mNSS scores were significantly lower in the CCI + 3D-MSCs group than in the CCI + ACSF group from 3 dpi onward, indicating improved overall neurological function. In the BWT, rats in the CCI + 3D-MSCs group showed shorter traversal times than those in the CCI + ACSF group at later time points (Figure 3B), consistent with improved motor coordination and balance. In contrast, hindlimb slip counts did not differ significantly between the two CCI groups (Figure 3C). Together, these findings indicate that intrathecal administration of 3D-MSCs promotes sensorimotor recovery after CCI.

### 3.4 3 D-MSCs improve long-term cognitive function after CCI

To assess spatial learning and memory, the MWM was performed at 30–34 dpi. The test consisted of a 4-day hidden-platform acquisition phase followed by a probe trial on day 5.

**FIGURE 4:**
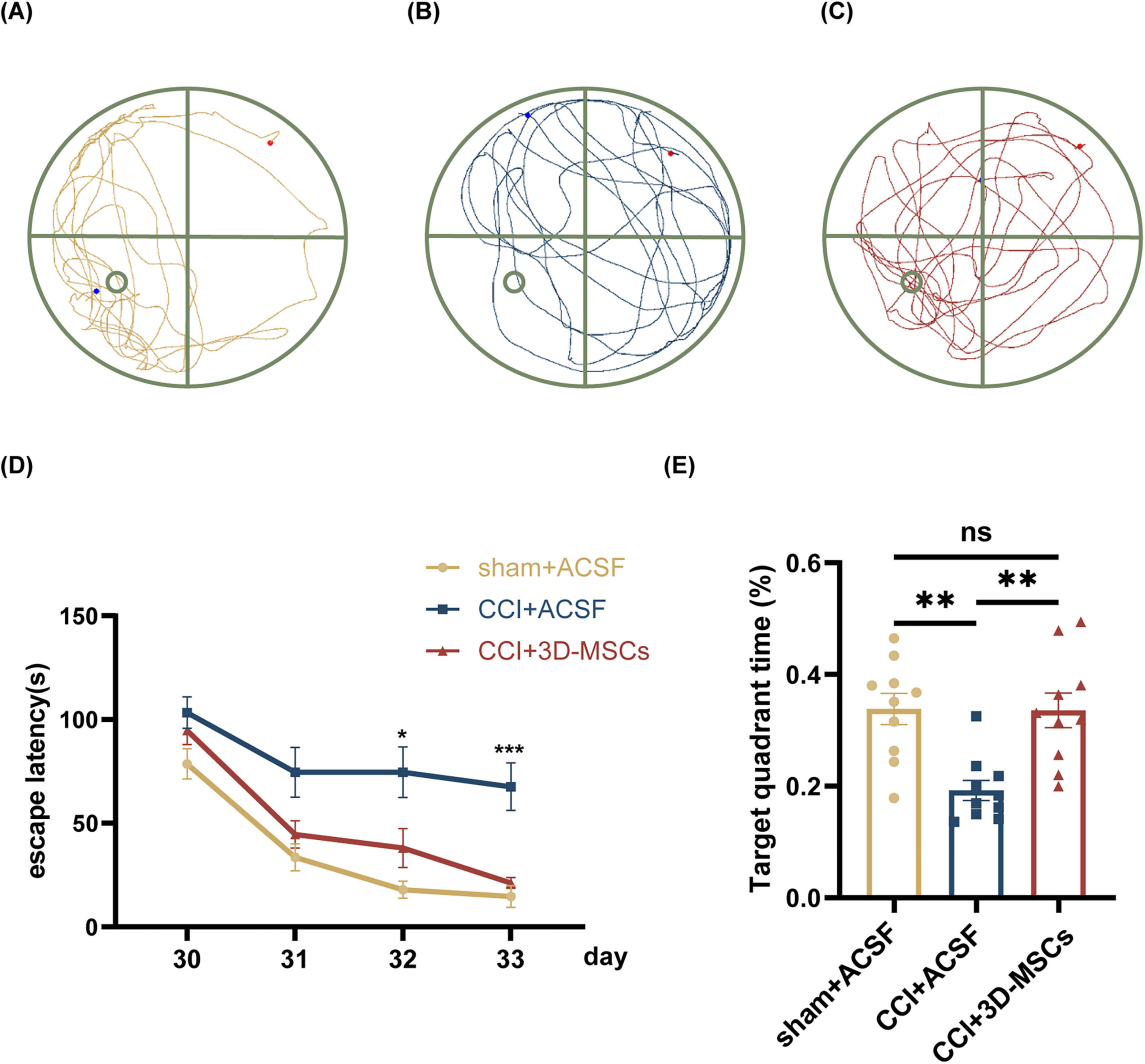
3D-MSCs improve long-term spatial learning and memory after CCI. **(A–C)** Representative swim paths from the Sham + ACSF, CCI + ACSF, and CCI + 3D-MSCs groups, respectively. The green circle indicates the platform location during the hidden-platform training phase. **(D)** Escape latency during the 4-day acquisition phase of the Morris water maze. **(E)** Proportion of time spent in the target quadrant during the probe trial. Data are shown as mean ± SD; *n* = 10 per group. **p < 0.01; ns, not significant. ACSF, artificial cerebrospinal fluid; CCI, controlled cortical impact; MSCs, mesenchymal stem cells.

During the acquisition phase, escape latency progressively decreased in all groups, indicating task learning over time (*p* = 0.008) (Figures 4A-D). *Post hoc* analysis showed that rats in the CCI + 3D-MSCs group had significantly shorter escape latencies than those in the CCI + ACSF group on day 3 (38.07 ± 29.56 s vs. 74.60 ± 38.50 s, *p* < 0.01) and day 4 (21.22 ± 8.05 s vs. 67.61 ± 36.17 s, *p* < 0.01).

In the probe trial, the proportion of time spent in the target quadrant differed significantly among groups (0.3384 ± 0.0886 in Sham + ACSF vs. 0.1922 ± 0.0572 in CCI + ACSF vs. 0.3359 ± 0.0984 in CCI + 3D-MSCs, *p* < 0.001) (Figure 4E). Rats in the CCI + 3D-MSCs group spent significantly more time in the target quadrant than those in the CCI + ACSF group (*p* = 0.002), whereas their performance was comparable to that of the Sham + ACSF group (*p* = 1.000).

Together, these findings indicate that intrathecal administration of 3D-MSCs at 24 h after CCI is associated with improved long-term spatial learning and memory.

### 3.5 3 D-MSCs attenuate widespread Iba1-associated neuroinflammatory changes after CCI

**FIGURE 5:**
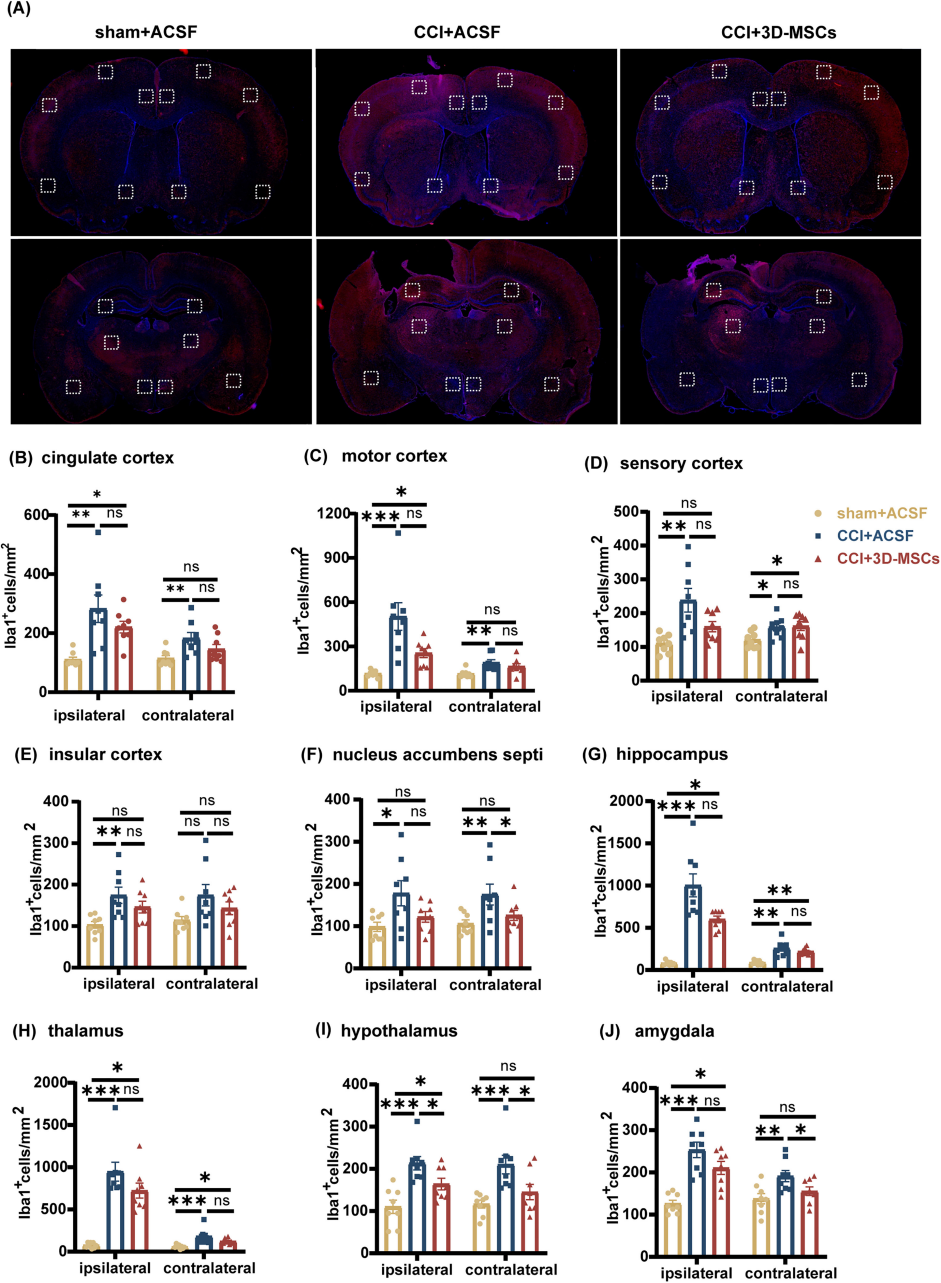
3D-MSCs attenuate widespread Iba1-associated neuroinflammatory changes after CCI. **(A)** Representative coronal brain sections illustrating the regions of interest (ROIs) selected for Iba1 immunofluorescence analysis. The upper panel shows coronal sections containing the cingulate cortex, motor cortex, sensory cortex, insular cortex, and nucleus accumbens septi. The lower panel shows coronal sections containing the hippocampus, thalamus, amygdala, and hypothalamus. White dashed boxes indicate the specific ROIs analyzed in this study. Iba1 staining is shown in red and DAPI in blue. **(B–J)** Quantification of Iba1-positive cell density in the bilateral cingulate cortex, motor cortex, sensory cortex, insular cortex, nucleus accumbens, hippocampus, thalamus, hypothalamus, and amygdala. Data are shown as mean ± SD; *n* = 4 per group. Two sections per animal were analyzed as technical replicates. *p < 0.05, **p < 0.01, ***p < 0.001; ns, not significant. ACSF, artificial cerebrospinal fluid; CCI, controlled cortical impact; Iba1, ionized calcium-binding adapter molecule 1; MSCs, mesenchymal stem cells.

To characterize the spatial distribution of post-traumatic neuroinflammatory changes and assess the immunomodulatory effects of treatment, brain tissues were collected at 7 dpi after intrathecal administration of 1 × 10^6^ 3D-MSCs or ACSF at 24 h after injury. Iba1-positive cells were quantified bilaterally in nine brain regions: the cingulate cortex, motor cortex, sensory cortex, insular cortex, nucleus accumbens septi, hippocampus, thalamus, hypothalamus, and amygdala (Figure 5; Supplementary Figures 1–9).

Compared with the Sham + ACSF group, the CCI + ACSF group showed marked increases in Iba1^+^ cell density across all examined bilateral brain regions, indicating that focal CCI was associated with widespread Iba1-associated neuroinflammatory changes throughout the brain at 7 dpi.

Importantly, intrathecal treatment with 3D-MSCs attenuated these changes. Compared with the CCI + ACSF group, the CCI + 3D-MSCs group showed significantly lower Iba1^+^ cell density in several regions, including the contralateral nucleus accumbens, ipsilateral hypothalamus, contralateral hypothalamus, and contralateral amygdala (all *p* < 0.05). Collectively, these findings suggest that intrathecal administration of 3D-MSCs at 24 h after CCI exerts broad immunomodulatory effects and attenuates disseminated Iba1-associated neuroinflammatory changes in both perilesional and remote brain regions.

## 4 Discussion

In this study, focal cortical injury induced by CCI was associated not only with local pathological changes in the perilesional cortex but also with widespread Iba1-associated neuroinflammatory changes in anatomically remote brain regions. Intrathecal administration of 3D-MSCs at 24 h after injury could migrate to the perilesional area in large numbers as early as 2 dpi. This migration corresponded with marked reduction of neuronal apoptosis in the ipsilateral brain region at 2 dpi, and decreased lesion volume at 7 dpi. In parallel, 3D-MSC treatment attenuated widespread Iba1-associated neuroinflammatory changes at 7 dpi. And a battery of behavioral assessments indicated that 3D-MSCs treatment significantly ameliorated comprehensive neurological dysfunction at the early stage, and improved cognitive deficits at 30 days following TBI.

Accumulating preclinical and clinical evidence indicates that both focal and diffuse traumatic brain injuries can trigger extensive neuroinflammatory responses that may persist for a long time (Shi et al., 2019; Bedi et al., 2023). Our findings further support this perspective. In a rat model of severe focal brain injury, significant increases in Iba1^+^ cell density were observed as early as 7 dpi across multiple bilateral brain regions, including the cingulate cortex, motor cortex, sensory cortex, insular cortex, nucleus accumbens septi, hippocampus, thalamus, hypothalamus, and amygdala. No such widespread changes were observed in the sham-operated group.

Notably, our data indicate that post-traumatic increases in Iba1^+^ cell density are regionally heterogeneous rather than uniformly distributed, with more prominent changes in deep and limbic regions, particularly the thalamus and hippocampus. The regional differences in Iba1^+^ expression observed after CCI may be interpreted from two complementary aspects. First, they likely reflect differences in the extent of direct mechanical injury and secondary injury processes. In our model, the cortical impact produces focal physical damage at the injury site, while secondary pathological cascades, including edema, excitotoxicity, oxidative stress, blood-brain barrier disruption, and delayed neurodegeneration, may further amplify neuroinflammation in adjacent or particularly vulnerable regions (Hajivalili et al., 2025). Therefore, brain areas located closer to the lesion or more susceptible to secondary injury may exhibit larger increases in Iba1 immunoreactivity or Iba1^+^ cell density, whereas other regions may show only modest changes at the same time point. Second, the observed pattern may also involve the propagation of injury-related and inflammatory signals to remote brain regions. Focal cortical injury can induce anterograde and retrograde Wallerian degeneration along anatomically connected white matter pathways, such as corticothalamic and corticostriatal projections, thereby extending neuroinflammation beyond the primary lesion site (Viscomi, 2020). This may be particularly relevant to the thalamus, given its dense reciprocal connections with the cortex, which may increase its vulnerability to transneuronal degeneration and secondary inflammatory activation after cortical trauma. Likewise, the hippocampus, a limbic structure highly sensitive to excitotoxicity, metabolic stress, and circuit disruption after TBI, may be especially prone to sustained microglial activation even without direct mechanical injury. diffusible inflammatory signals may help explain the more modest increases in Iba1^+^ reactivity observed in other remote regions. Inflammatory mediators, damage-associated molecular patterns, and pro-inflammatory cytokines released from the impact site may spread through the ventricular system, subarachnoid space, and glymphatic pathways, contributing to broader but less pronounced neurovascular and microglial responses (Gard et al., 2023). Together, these mechanisms may explain why the thalamus and hippocampus exhibited more robust inflammatory responses than other brain regions.

This study is, to our knowledge, the first to investigate the therapeutic efficacy of 3D-MSCs in a model of focal cortical trauma. By utilizing an intrathecal delivery route, the 3D-MSCs may exploit these same fluid dynamics. Introducing the cells directly into the CSF circulatory space bypasses the restrictive blood-brain barrier and may allow them to navigate via pathotropism toward diffuse chemoattractant gradients, thereby targeting both the cortical injury site and highly inflamed subcortical regions.

We initially characterized the homing kinetics of the 3D-MSCs, as targeted parenchymal engraftment represents an important prerequisite for downstream paracrine and structural repair. At 24 h post-intrathecal injection, the 3D-MSCs exhibited a highly selective, pathotropic migration toward the injured hemisphere, preferentially accumulating within the perilesional cortex while leaving intact tissue virtually uncolonized. Specifically, the density of 3D-MSCs on the injury side was approximately seven-fold higher than that on the contralateral side. Previous studies have suggested that the homing kinetics of stem cells may initiate earlier than the detection time points established in our experimental protocol. For instance, Kim et al. reported detectable stem cell engraftment within the brain as early as 12 h post-administration (Kim et al., 2020). Overall, these findings indicate that 3D-MSCs can rapidly and effectively target and aggregate at the lesion site, which may contribute to the initiation of therapeutic effects.

At the same time, the implications of this early localization for sustained recovery remain uncertain. Because transplanted 3D-MSCs were tracked only at 24 h after intrathecal administration, the present study demonstrates early lesion-directed homing but does not determine whether these cells persist at the injury site, migrate further, or are progressively cleared over the subsequent days and weeks. Therefore, the 24 h signal should be interpreted as evidence of early homing rather than sustained engraftment throughout the 34-day recovery period. Accordingly, although rapid perilesional accumulation may facilitate the initiation of therapeutic actions, our data do not establish the duration of cell residence, define the temporal window during which 3D-MSCs exert their effects, distinguish whether long-term functional benefits are mediated by sustained engraftment or transient paracrine/immunomodulatory signaling, or determine whether cell persistence correlates with the trajectory of functional recovery.

Consistent with prior intrathecal MSC-tracking studies in rodent CNS injury models (Supplementary Table 1), donor cells are generally detectable near lesions during the acute phase (approximately 1–7 days), decline substantially by 2–4 weeks, and are often undetectable by 4–8 weeks (Satake et al., 2004; Urdzikova et al., 2014; Krupa et al., 2018; Vives et al., 2022; Aguado-Garrido et al., 2024). Some reports have described intermediate detectability (approximately 4–6 weeks), usually with low retention and predominantly meningeal or perilesional rather than robust intraparenchymal localization (Bakshi et al., 2006; Paul et al., 2009; Cizkova et al., 2011). In a chronic TBI model, longer survival has been reported up to 6 months, although migration was sparse and the relevance to sustained efficacy remained unclear (Bonilla et al., 2014). Taken together, these studies suggest that durable engraftment after intrathecal delivery is generally limited. Thus, the long-term functional improvement observed in our study is more consistent with mechanisms initiated by early cell presence, including transient paracrine and immunomodulatory signaling, although a limited structural contribution cannot be excluded. Indeed, a few studies have reported partial neural-lineage differentiation of transplanted MSCs, including Nestin-positive progenitor-like differentiation in SCI (Satake et al., 2004), neuron-like differentiation signals in stroke (Lim et al., 2011), and similar observations in chronic TBI (Bonilla et al., 2014); however, these findings appear model-dependent and have not been consistently reproduced. Future longitudinal tracking studies, minimally including 7 dpi and 28 dpi, will be necessary to define 3D-MSC persistence kinetics, clarify the active therapeutic window, and determine how cell fate relates to functional recovery over time.

Our study showed that intrathecal administration of MSCs at 24 h after CCI significantly reduced apoptotic cells in the peri-lesional area at 2 dpi and improved neurological deficits at the early stage after TBI. MRI T2 also showed a trend toward smaller lesion volume at 2 dpi and a significant reduction at 7 dpi, and these therapeutic effects were accompanied by suppressed perilesional accumulation of Iba-1^+^ cells. Our findings support the view that MSCs confer early neuroprotection following TBI (Pischiutta et al., 2021). For instance, Zhang et al. (2024) reported that MSC therapy mitigates post-traumatic tissue loss and lesion expansion by modulating neuroinflammation and alleviating neuronal damage (Zhang et al., 2024). The delayed significance in lesion volume reduction further suggests that the protective effects of MSCs may first appear at the cellular level and later translate into structural protection. Collectively, our data support the view that MSCs exert early neuroprotective effects after TBI by reducing apoptosis and limiting lesion expansion. However, these early structural findings should be interpreted with caution when considering the later functional outcomes. Because MRI was performed only at 2 and 7 dpi, whereas cognitive function was assessed at 30–34 dpi, the early reduction in lesion volume and the later improvement in cognitive performance represent temporally dissociated observations that cannot be directly linked in the present study. Thus, we cannot determine whether the chronic cognitive benefit is related to persistent structural preservation, subsequent remodeling, or other therapeutic mechanisms not captured by the current imaging schedule.

It is worth noting that the temporal profiles of behavioral recovery were not identical across assessments. While mNSS consistently showed significant differences between the CCI groups throughout the observation period, the average traversal time in the beam walk test differed only at later time points, and the number of hind limb slips remained comparable between groups. Such discrepancies likely reflect differences in the functional domains and sensitivity of the tests. As a composite neurological score encompassing motor, sensory, reflex, and balance functions, mNSS may be more sensitive to early and global neurological changes after TBI. By contrast, beam walk-derived measures assess more specific aspects of coordinated locomotion and postural control. In the early post-injury stage, prominent limb weakness or gross motor impairment may exert a dominant influence on beam walk performance, thereby obscuring more subtle between-group differences in balance and fine motor coordination. As motor disability gradually improves during later stages of recovery, deficits in more refined functions, such as balance control and coordinated locomotion, may become more readily detectable. The absence of significant differences in hind limb slips may further suggest that this parameter is relatively insensitive in our model or more strongly influenced by inter-animal variability and compensatory motor strategies. This interpretation is consistent with a systematic review and meta-analysis (Pischiutta et al., 2021), which showed that although MSC therapy improves sensorimotor outcomes in preclinical TBI models, the sensitivity and variability differ across behavioral assessments. Taken together, these findings suggest that the divergence among behavioral readouts in our study more likely reflects differences in test responsiveness and stage-dependent functional recovery than inconsistency in the neurobehavioral effects of MSC treatment.

Beyond the early protective effects on the injury site, 3D-MSC transplantation significantly suppressed widespread Iba1-associated neuroinflammatory changes during the subacute phase of TBI. On postoperative day 7, vehicle-treated CCI rats exhibited disseminated Iba1-associated neuroinflammatory changes across multiple brain regions, which were significantly attenuated by a single intrathecal delivery of 3D-MSCs. Consistent with these findings, Bedi et al. also confirmed in a severe CCI rat model that bone marrow mononuclear cell treatment significantly suppressed microglial activation in the ipsilateral cerebral hemisphere, as quantified *in vivo* using standardized uptake values of the positron emission tomography ligand [18F] PBR28, which targets the translocator protein—a clinically used marker of neuroinflammation imaging. Their histological analysis showed that bone marrow mononuclear cell administration also markedly reduced the number of amoeboid microglia in the hippocampal dentate gyrus region, consistent with our finding of lowered Iba1^+^ cell density across multiple brain regions (Bedi et al., 2023). The aforementioned findings suggest that 3D-MSCs significantly reduce Iba1^+^ cell accumulation and attenuate associated neuroinflammatory changes in the injury core and multiple distant brain regions. These 7-dpi inflammatory findings should be interpreted as subacute histological observations and should not be taken as evidence that the same inflammatory changes persisted into the chronic phase when cognitive function was evaluated.

In our preliminary study, 3D-MSC treatment improved cognitive performance at 30–34 dpi, indicating a beneficial effect on long-term functional outcome after TBI. This delayed behavioral improvement is an important finding, as it suggests that intrathecal MSC administration may confer sustained therapeutic benefit beyond the acute stage of injury. Previous studies have suggested several mechanisms through which MSC-based interventions may influence recovery after CNS injury (Zhang et al., 2013; Pischiutta et al., 2021; Qin et al., 2022; Ferreira et al., 2026). However, whether any of these mechanisms account for the late cognitive improvement observed in the present study remains unknown. In particular, neuroinflammatory changes were assessed only at 7 dpi, whereas cognitive performance was evaluated nearly 4 weeks later. Therefore, the anti-inflammatory effect observed at 7 dpi and the cognitive benefit detected at 30–34 dpi should be interpreted as temporally dissociated observations, and no direct causal relationship between them can be established based on the current data. Although early modulation of the post-traumatic environment may potentially be relevant to later functional recovery, this possibility remains speculative in the absence of longitudinal structural and inflammatory assessments across the chronic stage. Future studies incorporating later time points will be necessary to clarify the mechanisms underlying the delayed cognitive benefit of MSC treatment.

**FIGURE 6:**
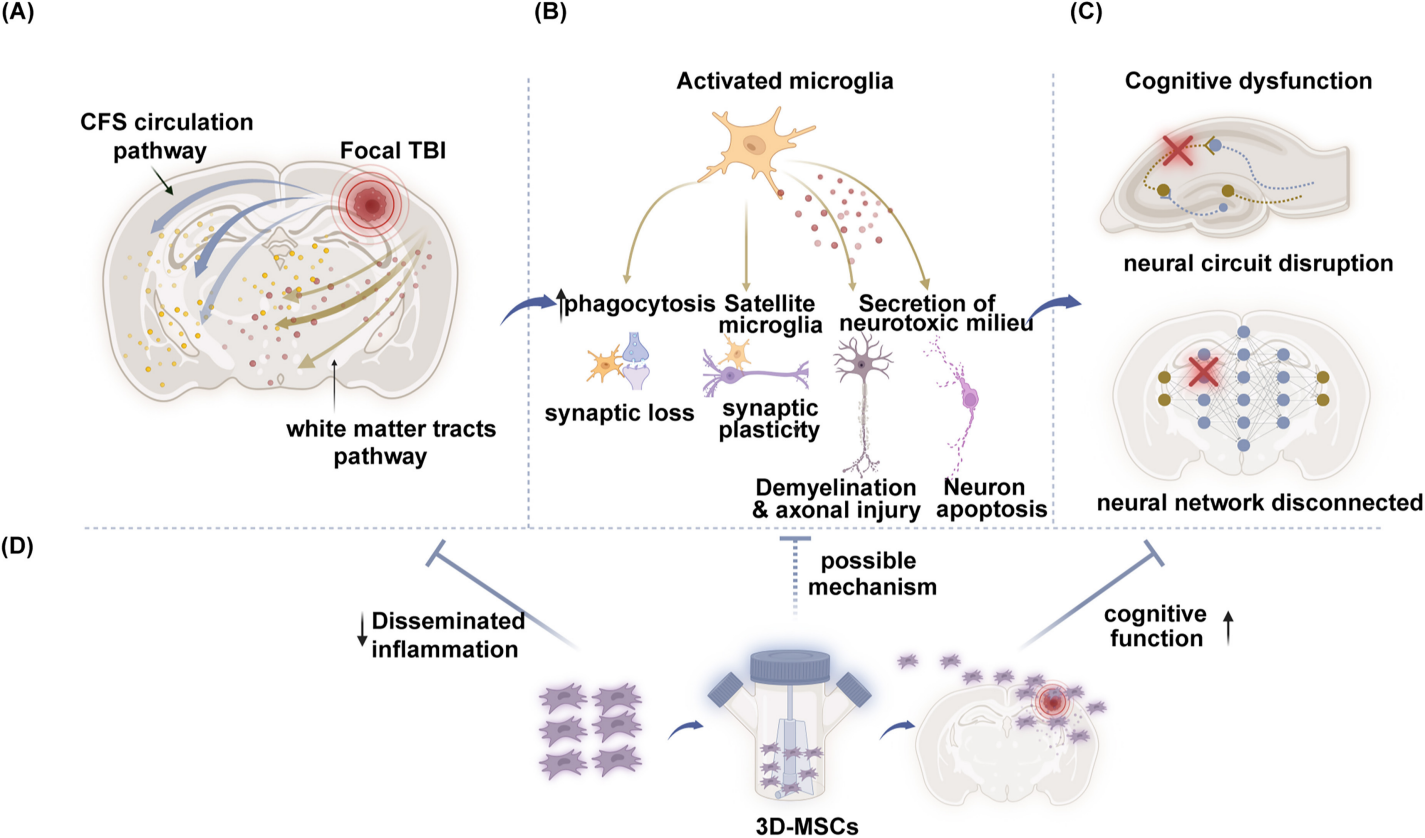
Hypothetical conceptual framework for the possible effects of 3D-MSC treatment after TBI. **(A)** Focal TBI may trigger widespread Iba1-associated changes extending beyond the primary lesion, potentially through CSF-mediated and white matter-associated routes. **(B)** Such diffuse neuroinflammatory responses may be related to microglia-associated processes including altered phagocytic activity, satellite microglia formation, release of inflammatory mediators, synaptic alterations, axonal injury, and neuronal vulnerability. **(C)** These processes may potentially affect neural circuit organization and broader brain network connectivity, thereby being relevant to later cognitive impairment. **(D)** Intrathecally administered 3D-MSCs were observed near the injury site and were associated with reduced widespread Iba1-associated changes at 7 dpi and improved cognitive performance at 30–34 dpi. However, the mechanistic relationships illustrated here were not directly tested and should be regarded as a speculative working hypothesis. MSCs, mesenchymal stem cells; TBI, traumatic brain injury; CSF, cerebrospinal fluid.

Overall, the present study should be considered a proof-of-concept investigation. Based on our findings, together with previous literature (Krukowski et al., 2021; Feichtenbiner et al., 2025; Li et al., 2025; Santurro et al., 2025), we cautiously propose as a working hypothesis that early modulation of the post-traumatic inflammatory environment may be one of several processes potentially relevant to later cognitive outcome after TBI (Figure 6). At present, however, this remains a hypothesis, as the mechanistic relationship was not directly examined in our study. This hypothesis requires direct validation in future studies incorporating longitudinal inflammatory assessments, chronic imaging, and synaptic or circuit-level analyses.

Nevertheless, this study has several limitations. First, a conventional 2D-MSC treatment group was not included; therefore, although 3D-MSCs were selected based on prior evidence suggesting enhanced therapeutic potential, the present study could not directly determine whether 3D-MSCs are superior to 2D-MSCs in the CCI model. Second, MRI was performed only at 2 and 7 days post-injury, with no imaging data available at later time points such as 14 or 28 dpi. Therefore, the present study provides no direct evidence linking early lesion-volume reduction to chronic cognitive improvement. Third, because the histopathological analysis relied mainly on Iba1 staining, we could not definitively distinguish resident microglia from infiltrating macrophages or characterize their activation states. Future studies incorporating additional markers, such as TMEM119, P2RY12, CD68, Ki67, iNOS, or Arg1, are needed. Fourth, the fate of transplanted 3D-MSCs was assessed only at 24 h post-transplantation; therefore, the temporal relationship between early cell localization and long-term therapeutic outcomes could not be established. Future studies incorporating longitudinal cell-tracking approaches are needed to define the *in vivo* persistence of transplanted 3D-MSCs and its relationship to functional recovery.

## 5 Conclusion

In conclusion, this proof-of-concept study shows that intrathecal administration of 3D-MSCs after CCI is associated with early lesion-directed homing, reduced acute neuronal apoptosis and subacute lesion burden, attenuated widespread Iba1-associated neuroinflammatory changes, and improved neurological and cognitive outcomes after TBI. These findings support the therapeutic potential of 3D-MSCs for experimental focal cortical injury, while further longitudinal studies are warranted to clarify the mechanisms underlying sustained functional recovery.

## Data Availability

The original contributions presented in this study are included in this article/Supplementary material, further inquiries can be directed to the corresponding authors.
