# Supplementary material for: Intrathecal injection of 3D-mesenchymal stem cells attenuates disseminated neuroinflammation and improves cognitive function in controlled cortical impact rats
Source: Front Neurosci. 2026 Jul 28;20:1895538. doi: 10.3389/fnins.2026.1895538 (PMC13457469; doi:10.3389/fnins.2026.1895538)
Supplement: Supplementary file 1 [file Data_Sheet_1.pdf]

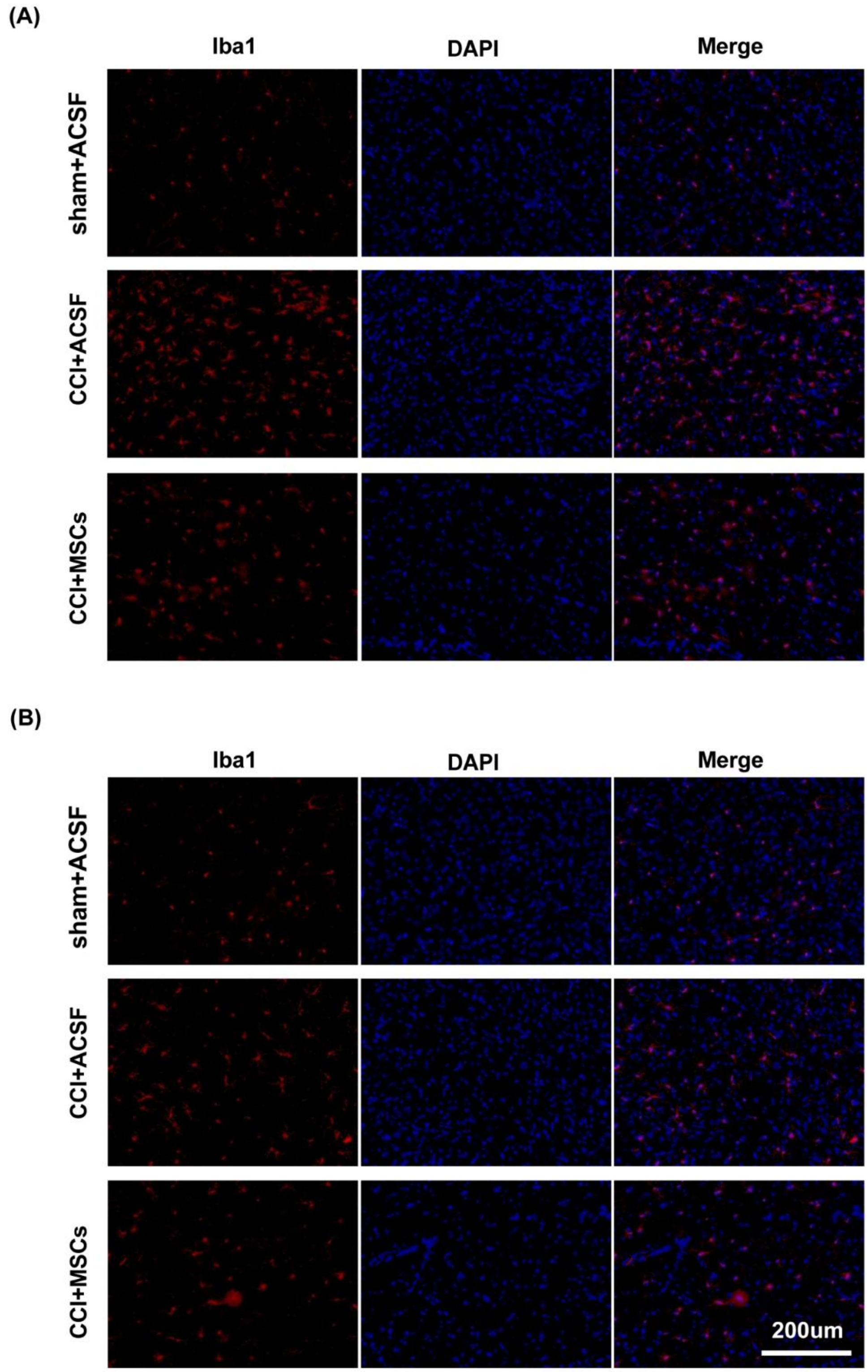

Supplementary Figure 1. IBA1, DAPI and merged immunofluorescence images of the cingulate cortex. (A) Ipsilateral region; (B) Contralateral region.

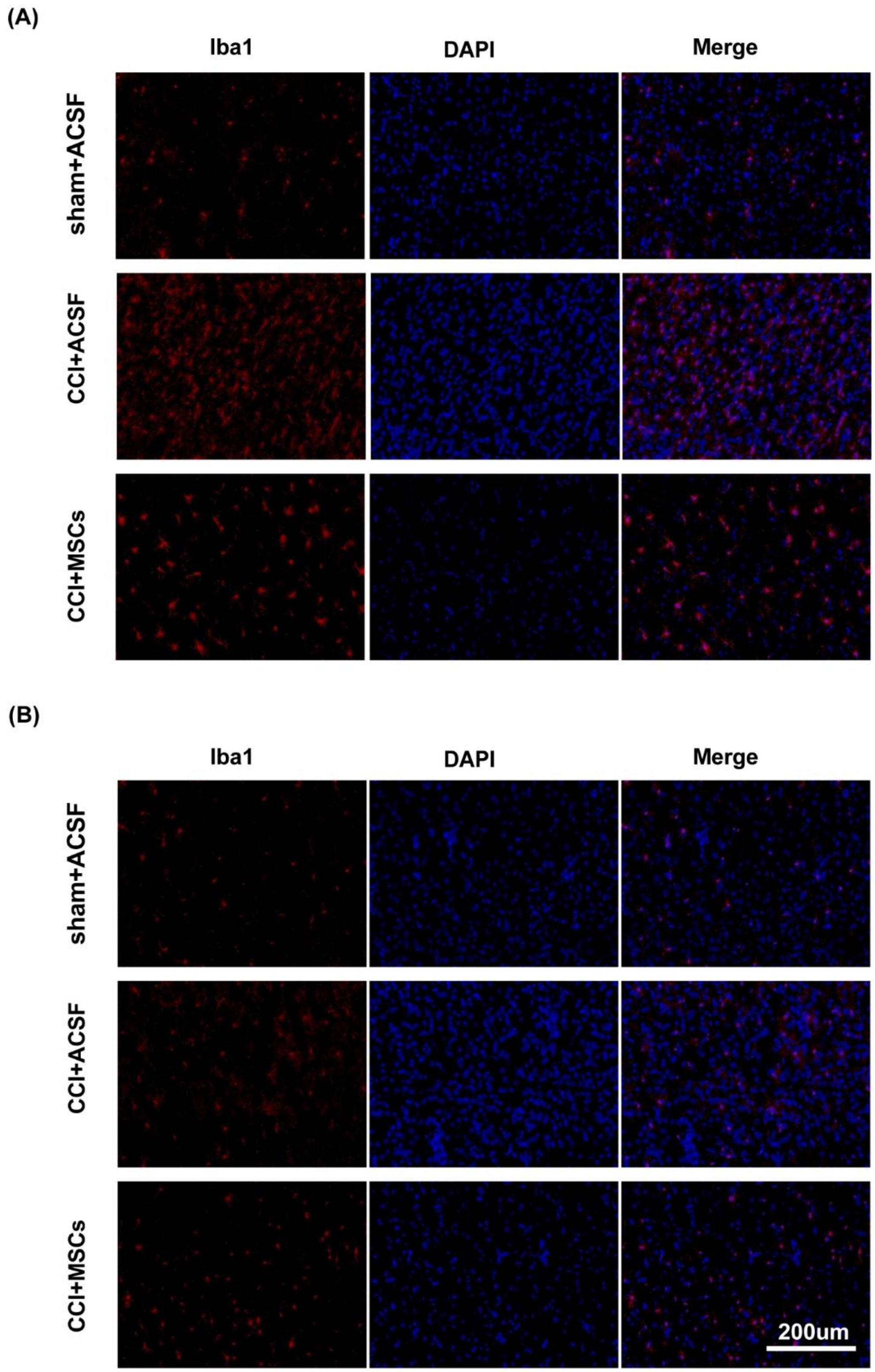

Supplementary Figure 2. IBA1, DAPI and merged immunofluorescence images of the motor cortex. (A) Ipsilateral region; (B) Contralateral region.

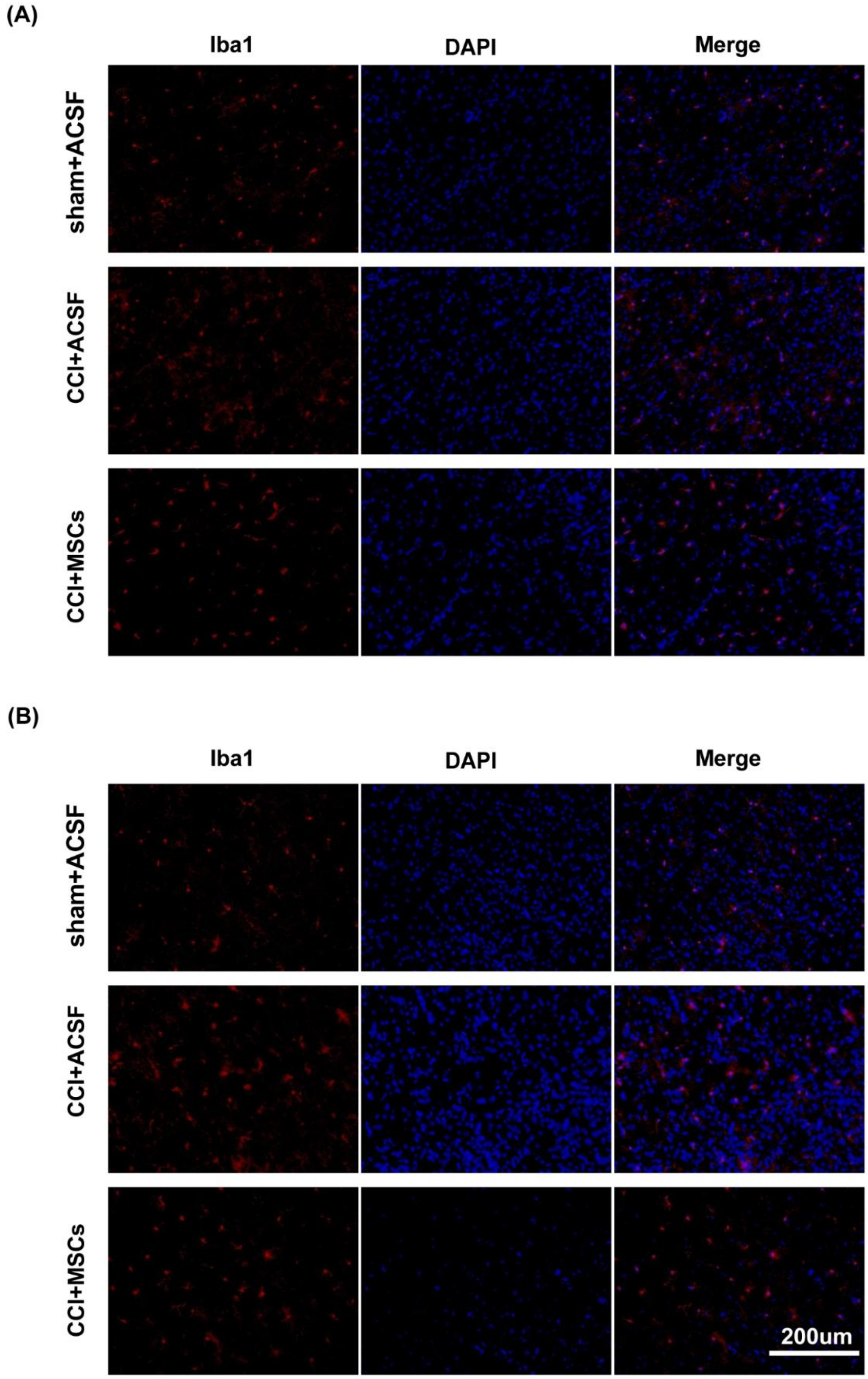

Supplementary Figure 3. IBA1, DAPI and merged immunofluorescence images of the sensory cortex. (A) Ipsilateral region; (B) Contralateral region.

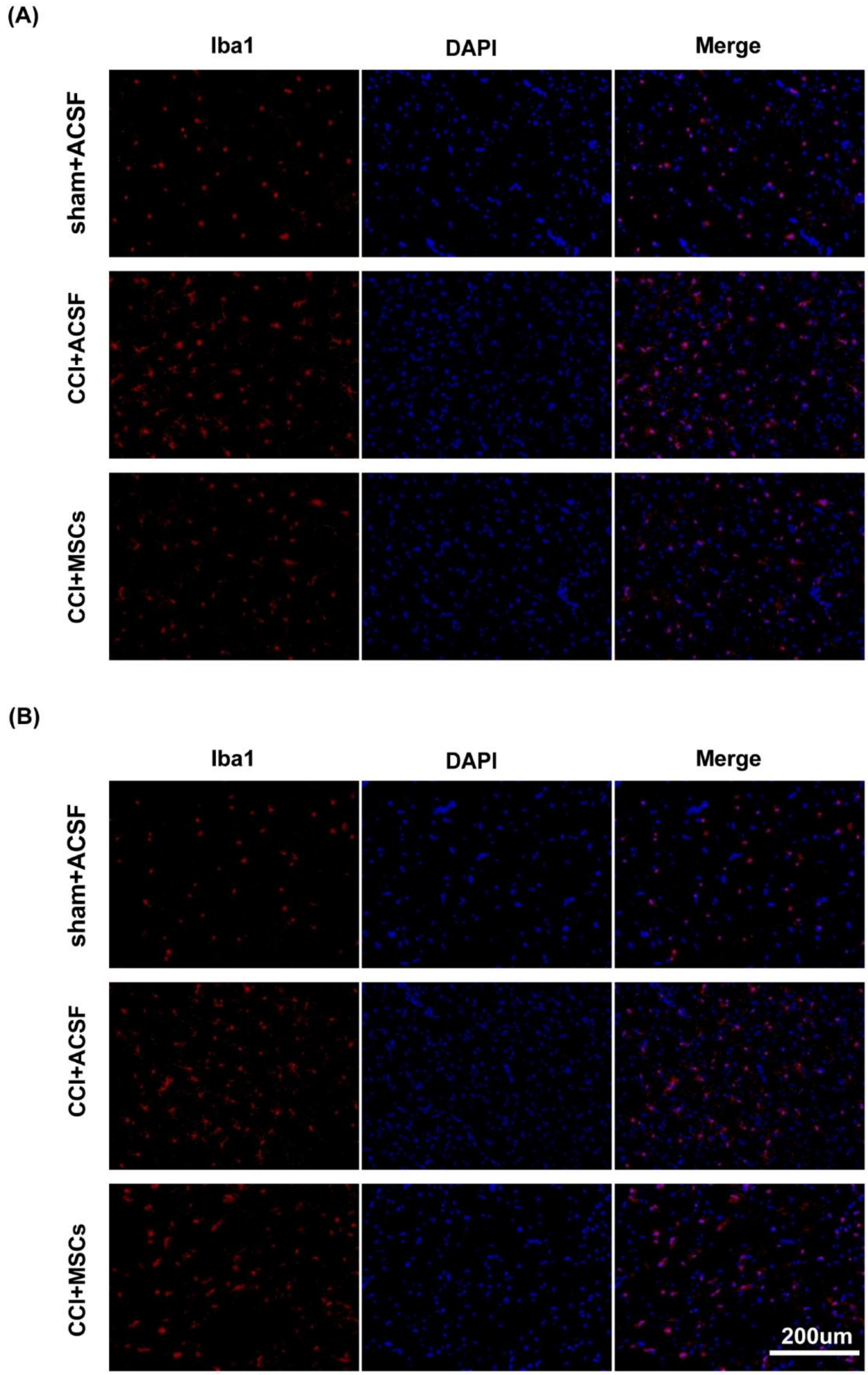

Supplementary Figure 4. IBA1, DAPI and merged immunofluorescence images of the insular cortex. (A) Ipsilateral region; (B) Contralateral region.

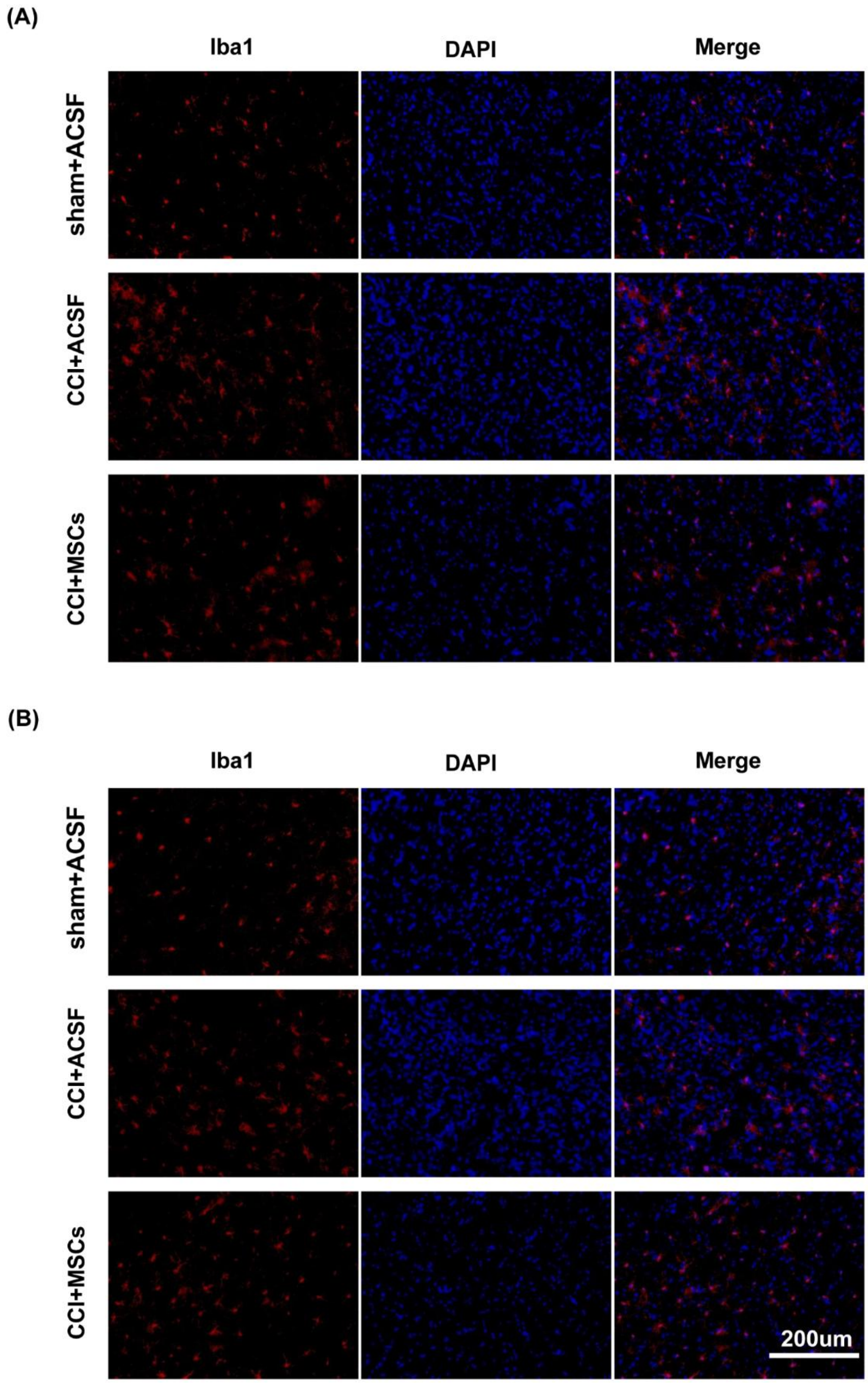

Supplementary Figure 5. IBA1, DAPI and merged immunofluorescence images of the nucleus accumbens septi. (A) Ipsilateral region; (B) Contralateral region.

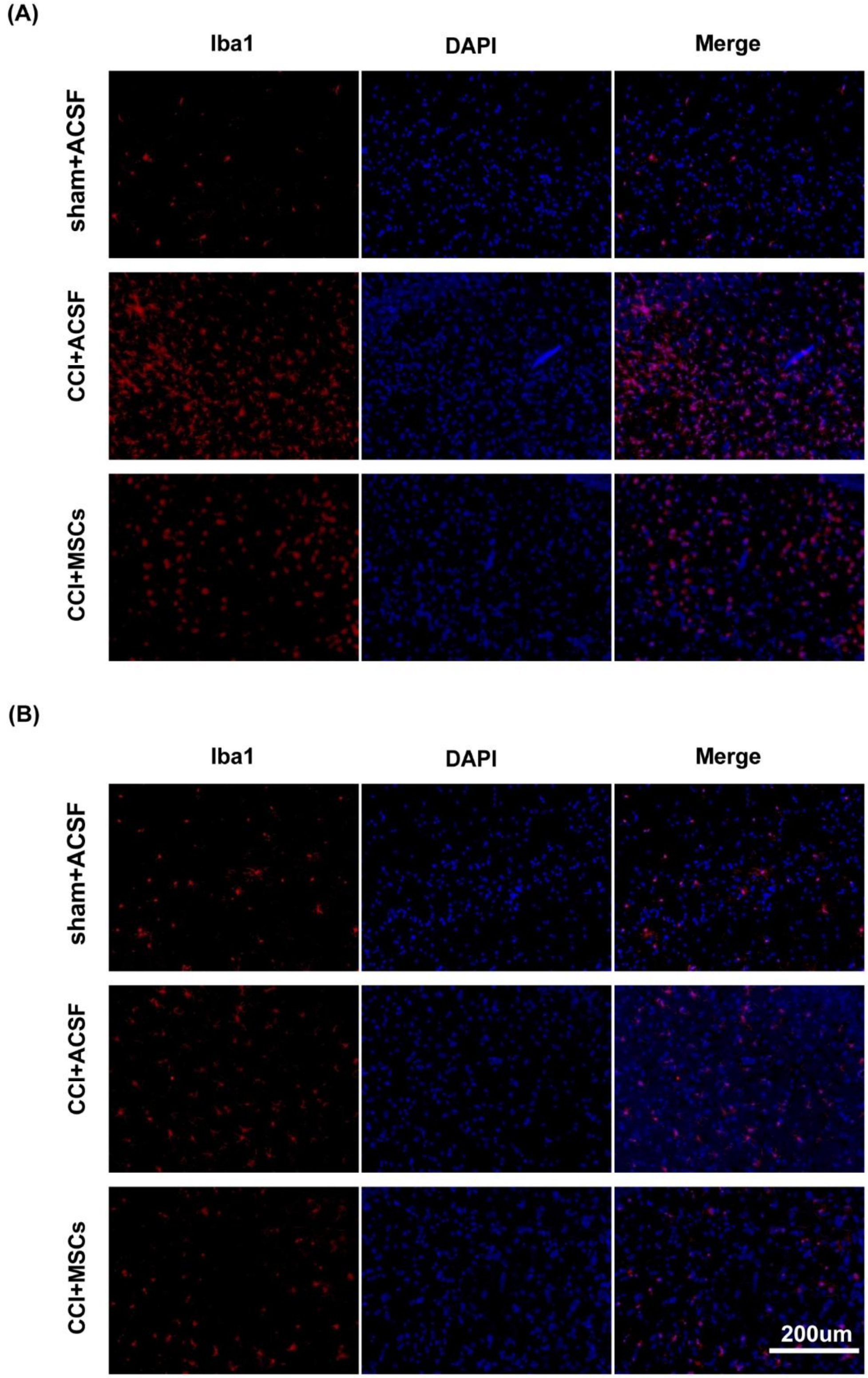

Supplementary Figure 6. IBA1, DAPI and merged immunofluorescence images of the hippocampus. (A) Ipsilateral region; (B) Contralateral region.

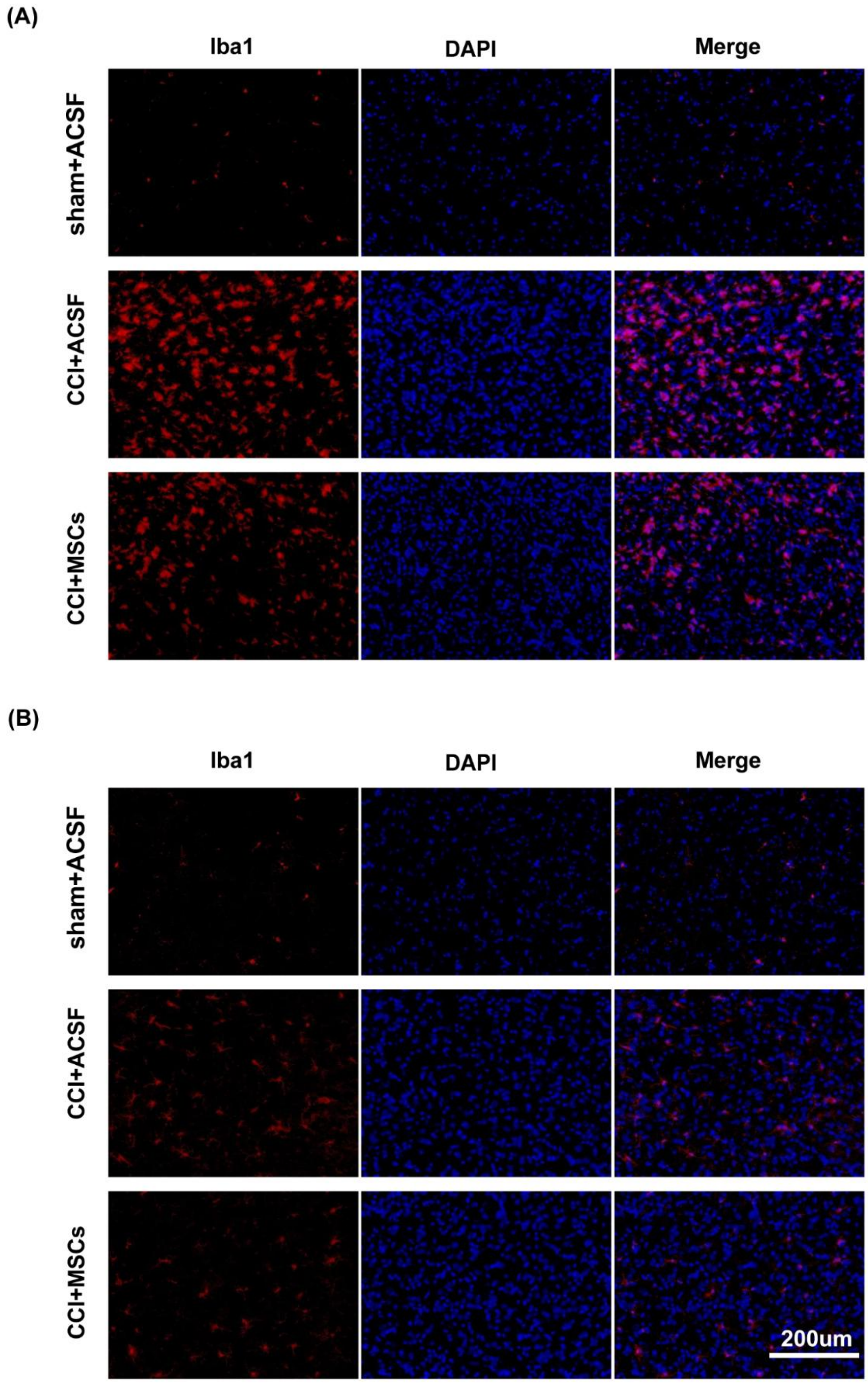

Supplementary Figure 7. IBA1, DAPI and merged immunofluorescence images of the thalamus. (A) Ipsilateral region; (B) Contralateral region.

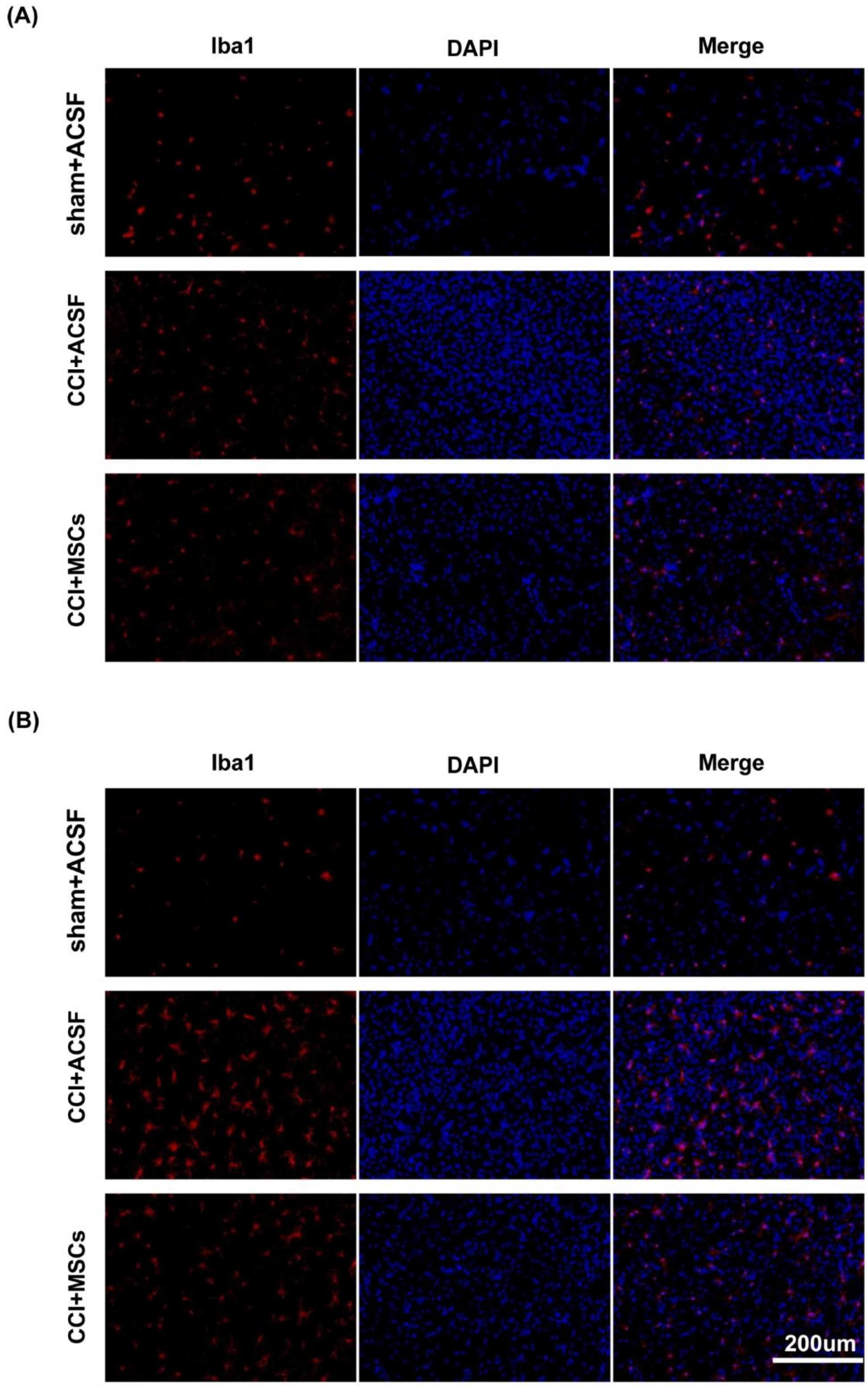

Supplementary Figure 8. IBA1, DAPI and merged immunofluorescence images of the hypothalamus. (A) Ipsilateral region; (B) Contralateral region.

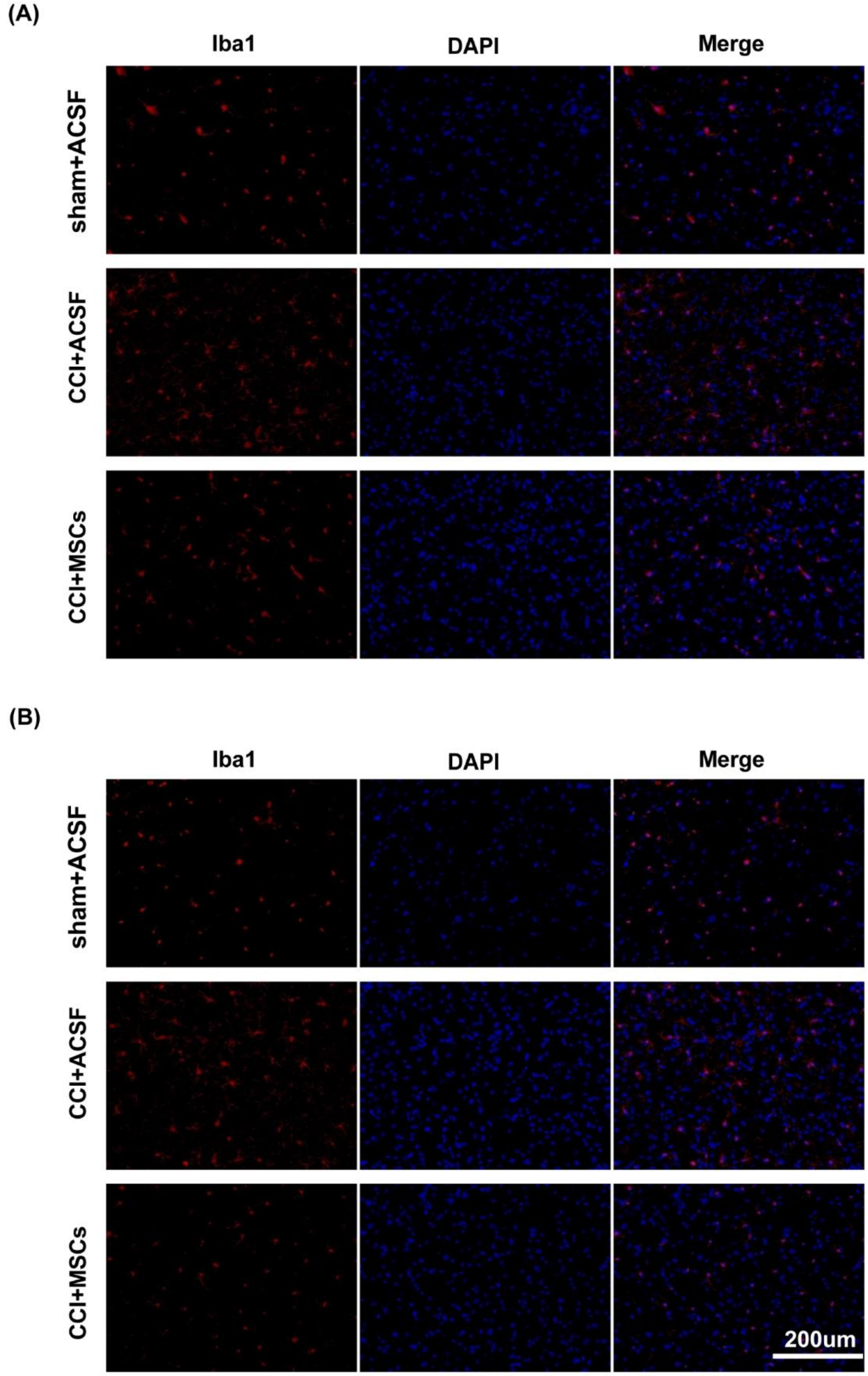

Supplementary Figure 9. IBA1, DAPI and merged immunofluorescence images of the amygdala. (A) Ipsilateral region; (B) Contralateral region.
