# Supplementary material for: Intrathecal injection of 3D-mesenchymal stem cells attenuates disseminated neuroinflammation and improves cognitive function in controlled cortical impact rats
Source: Front Neurosci. 2026 Jul 28;20:1895538. doi: 10.3389/fnins.2026.1895538 (PMC13457469; doi:10.3389/fnins.2026.1895538)
Supplement: Supplementary file 2 [file Table_1.pdf]

## Supplementary Table 1 MSC survival kinetics following intrathecal administration in rodent CNS injury models

| Disease | Author (Year) | Species | MSC source | Cell label | Transplantation timing | Evaluation time points (post-transplant) | Persistence result | MSC localization | Functional outcome | Mechanistic interpretation |
|---|---|---|---|---|---|---|---|---|---|---|
| SCI | Satake et al. (2004) | rat | Rat BM-MSC | GFP immunostaining | 3/5/7 dpi | 7 d, 14 d, 28 d | High early detectable signal; reduced at 14 d; undetectable by 28 d | Subarachnoid accumulation near lesion; partial perivascular entry into parenchyma | no proven long-term motor benefit | MCP-1 chemotaxis mediates migration; cells invade parenchyma via perivascular spaces; early inflammatory microenvironment impairs survival; partial Nestin⁺ progenitor differentiation |
| SCI | Bakshi et al. (2006) | rat | Rat BM stromal cells (AP-transgenic donor labeling) | AP histochemistry+ IF | 7 dpi | 1 w, 2 w, 6 w | Detectable through 6 weeks | Aggregated along dorsal lesion surface/impact point; limited parenchymal penetration | Behavior not primary endpoint (histology-focused study) | Paracrine trophic tissue protection |
| SCI | Paul et al. (2009) | rat | Human BM-MSC | HuNu IHC | 1 dpi | 4 d, 21 d | Detectable; declined from 4.1% (d4) to 3.4% (d21) | Mainly accumulated in injured white matter; no migration to rostral/caudal spinal or brain | better tissue sparing, less glial scar and immune infiltration than IV/local injection | Paracrine neurotrophic factors drive neuroprotection |
| SCI | Pal et al. (2010) | rat | Human BM-MSC | PKH26 fluorescence + histology | 3 or 14 dpi | 4 w | Detectable | Concentrated at lesion; no residual cells at lumbar injection site | Dose-dependent improvement in motor, sensory and balance function; 3 d vs 14 d transplantation shows no efficacy difference | Paracrine action dominant, no neural transdifferentiation |
| SCI | Cizkova et al. (2011) | rat | Rat BM-MSC | PKH67 fluorescence + histology | Single injection (3 or 7 dpi) or repeated daily injections (3–5 or 7–9 dpi) | 28 d | Detectable at 28 d with low overall retention; best group ~4.67% survival | Repeated dosing enabled migration/integration in central lesion white matter; single dose mainly pia/anterior spinal artery region | Single/early repeated dosing yields no significant functional gain; repeated dosing at 7–9 dpi significantly improves BBB scores up to 28 d | Acute inflammation causes massive graft apoptosis; engraftment improves as inflammation subsides; repeated dosing sustains paracrine supply; benefit is paracrine rather than cell replacement |
| SCI | Vaněček et al. (2012) | rat | Rat BM-MSC (GFP-transgenic) | GFP fluorescence | 1 wpi | 12 h, 24 h, 72 h, 168 h | Peak at 24 h; continuous decline; low at 168 h | Subarachnoid diffusion along cord with lesion-area enrichment | NA | NA |
| SCI | Urdzíková et al. (2014) | rat | Human BM-MSC | CFDA-SE; human nuclei/mitochondria markers | 7 dpi | 1 w and 8 w | Minimally detectable; undetectable at 8 weeks | Mostly intrathecal/dorsal meningeal attachment; minimal/no robust parenchymal integration | Single injection induces sustained motor and balance improvement without allodynia; attenuates glial scar | Short-term paracrine immunomodulation |
| SCI | Wang et al. (2014) | rat | Rat BM-MSC | DsRed lentiviral labeling | 7 dpi | 35 d | Detectable | Perilesional distribution; deep integration not clearly shown | NT-3-modified MSCs yield best motor recovery, smallest cyst cavity, most axonal regeneration and weakest glial scar | NT-3 enhances MSC survival and paracrine secretion; upregulates endogenous BDNF/VEGF; inhibits glial scar and promotes axonal regeneration |
| SCI | Zhang et al. (2015) | rat | Rat BM-MSC | SPIO nanoparticle labeling | 7 dpi | 1 d, 2 d, 4 d | Slight increase at 1–2 d, marked decay by 4 d | CSF-dominant spread; only small fraction retained near lesion | mildly improves motor function and axonal regeneration | Un-directed cells dilute and wash out in CSF, leading to insufficient lesion engraftment and limited repair efficacy |
| SCI | Ruzicka et al. (2017) | rat | Human BM-MSC | CFDA-SE | 7 dpi | 2 w, 2 m | Few cells at 2 w; none at 2 m | Attached to dorsal surface only; no parenchymal invasion | Improves motor function, no allodynia; limited axonal regeneration and tissue protection | Exclusively paracrine action, no neural differentiation or parenchymal integration; short-term anti-inflammatory and trophic effects; limited long-term repair potential |
| SCI | Krupa et al. (2018) | rat | Human WJ-MSC | HuNu IF | Single injection (7 dpi) or repeated injection (7+14+21 dpi) | 2 w, 4 w, 9 w | Short survival (2 w); no detectable donor cells in later windows (4/9 w) | Mostly retained in arachnoid folds/cauda equina sheath; poor intraparenchymal homing | Efficacy depends on dose and dosing frequency; repeated dosing enables weight-bearing locomotion and superior fine motor function | Paracrine effects |
| SCI | Mohamadi et al. (2019) | rat | Human WJ-MSC | CM-DiI fluorescence | 48 h post-injury | 1 w | Consistently detected | Lesion centered, mainly beneath the pia mater and within the posterior fasciculus | Improved motor function; protects ventral horn neurons | Inhibits NLRP1 inflammasome activation and downstream proinflammatory cytokines; paracrine PGE2/TSG-6 suppress neuroinflammation and protect neurons |
| SCI | Sun et al. (2019) | rat | Human UC-MSC | HuNu IF | 2 wpi | 2 w | Detectable | Primarily around lesion cavity/glial scar border; limited spread | UC-MSC monotherapy also improves function | UC-MSCs act via paracrine, no neural differentiation |

| | | | | | | | | | | |
|---|---|---|---|---|---|---|---|---|---|---|
| SCI | Romero-Ramírez et al. (2020) | rat | Human BM-MSC | Human mitochondrial Cy3 IF | 1–2 h post-injury | 10 w | Undetectable | NA | Acute injection outperforms methylprednisolone in early motor function, no difference vs saline at 9 weeks; no allodynia, no tissue repair effect | Inhibits microglia/macrophage activation and neuronal apoptosis; paracrine neuroprotection via extracellular vesicles |
| SCI | Yang et al. (2020) | rat | Human UC-MSC | Luciferase BLI + HNA/Ku80 IF | 10 dpi | BLI (1d , 3 d, 5 d, 7 d); histology (30 d) | BLI signal absent by day 7; small residual donor cells at day 30 histology | Mostly confined to spinal canal; limited homing into lesion parenchyma | Improves motor function, shortens bladder recovery, enhances electrophysiological and imaging outcomes; reduces cavity size, glial scar and increases myelin/neurons | Minor glial differentiation supports remyelination; paracrine immunosuppression and endogenous progenitor activation; improves neural conduction |
| SCI | Vives et al. (2022) | rat | Human WJ-MSC | Anti-human mitochondria (short-term), Stem101 (mid/long-term), human CART PCR (Systemic distribution) | Single injection (7 dpi) or repeated injection (7+14 dpi) | 2 d, 14 d, 70 d after dosing | Detectable at 2 d; undetectable at 14 and 70 d | Early lesion-proximal signal in spinal cord; no long-term organ retention | Double dosing yields better motor function, electrophysiology and tissue sparing than single dose | Efficacy entirely paracrine, no cell replacement; |
| SCI | Cao et al. (2022) | rat | Human UC-MSC | GFP fluorescence | 7 dpi | 30 d | Undetectable | No sustained lesion-center engraftment | Medium/high doses improve motor and electrophysiological function, low dose ineffective; reduces lesion cavity, promotes myelin and axon regeneration | Paracrine |
| SCI | Liu et al. (2023) | rat | Human UC-MSC | No dedicated fluorescent label; human-specific gene detection (qPCR) | 24 h post-injury | 28 d | Undetectable; transient lesion-homing trend at higher dose | No stable long-term integration | Dose-dependent motor improvement and lesion volume reduction | Paracrine |
| SCI | Aguado-Garrido et al. (2024) | rat | Human BM-MSC | Human mitochondrial IF | 7 dpi | 1 d, 2 d, 4 d, 7 d, 6 w | Detectable only at day 1; absent thereafter | Perimeningeal distribution near dorsal lesion segment | Improves motor coordination and balance, no effect on pain threshold | Short-term paracrine action, no long-term engraftment |
| TBI | Bonilla et al. (2014) | rat | Rat BM-MSC | Sry in situ hybridization | 2 months post-TBI | 6 m | Long-term survival up to 6 months (model-specific exception) | Primarily retained along spinal subarachnoid route; sparse migration to brain lesion border | Behavioral and histological improvements do not reach statistical significance | Cells migrate to brain lesion via CSF and can differentiate into neural cells; insufficient chemotactic signals in chronic phase lead to low engraftment and limited efficacy |
| Ischemic Stroke | Lim et al. (2011) | rat | Human UC-MSC | HuNu IF | 3 dpi | 7 d, 28 d | Detectable at least to 28 d | Restricted to ipsilateral ischemic boundary zone; absent contralateral hemisphere | motor improvement and infarct reduction | partial cells differentiate into mature neurons; induces neurogenesis and angiogenesis |
| Ischemic Stroke | Xu et al. (2025) | rat | Human UC-MSC | RFP lentiviral label | Single injection (postnatal day (P) 21) or repeated injection (P21/28/35/42) | 3 d, 7 d | Clearly detectable at 3 d; very low by 7 d | Signals in cortex and striatum injury territories | Repeated dosing outperforms single dose in motor function, myelin repair and inflammation suppression | Repeated dosing sustains chronic inflammation inhibition, glial scar attenuation, remyelination and trophic support |
| Ischemic Stroke | Huang et al. (2026) | rat | Human UC-MSC | HuNu IF | 72 h post-injury | 14 d, 28 d | Detectable at 14 and 28 d with time-dependent decline | Restricted to ipsilateral peri-infarct zone; absent contralateral hemisphere | improve neurological function and reduce infarct volume; improve neurological function and reduce infarct volume | Inhibits neuroinflammation and glial activation; promotes angiogenesis and endogenous neurogenesis; BDNF upregulation mediates neuroprotection and plasticity |

## Abbreviations

AP = alkaline phosphatase; BBB = Basso, Beattie, Bresnahan locomotor rating scale; BDNF = brain-derived neurotrophic factor; BLI = bioluminescence imaging; BM-MSC = bone marrow-derived mesenchymal stem cell; CFDA-SE = 5(6)-carboxyfluorescein diacetate succinimidyl ester; CM-DiI = chloromethylbenzamido-DiI; CNS = central nervous system; CSF = cerebrospinal fluid; Cy3 = cyanine 3; d = day; dpi = days post-injury; DsRed = Discosoma sp. red fluorescent protein; GFP = green fluorescent protein; HNA = human nuclear antigen; hWJ-MSC = human Wharton's jelly MSC; HuNu = human nuclei; IF = immunofluorescence; IHC = immunohistochemistry; IV = intravenous; Ku80 = Ku80 autoantigen; m = month; MCP-1 = monocyte chemoattractant protein-1; MSC = mesenchymal stem cell; NA = not applicable; NLRP1 = NLR family pyrin domain containing 1; NT-3 = neurotrophin-3; PCR = polymerase chain reaction; PGE2 = prostaglandin E2; PKH26 = PKH26 fluorescent cell linker; PKH67 = PKH67 fluorescent cell linker; qPCR = quantitative polymerase chain reaction; RFP = red fluorescent protein; SCI = spinal cord injury; SPIO = superparamagnetic iron oxide; Sry = sex-determining region Y; TBI = traumatic brain injury; TSG-6 = tumor necrosis factor-stimulated gene 6; UC-MSC = umbilical cord-derived mesenchymal stem cell; VEGF = vascular endothelial growth factor; w = week; WJ-MSC = Wharton's jelly MSC; wpi = weeks post-injury.

## Supplementary References


Aguado-Garrido, M., Garcia-Rama, C., Romero-Ramirez, L., Buzoianu-Anguiano, V., Perez-Rizo, E., Kramer, B.W., et al. (2024). Improved Efficacy of Delayed Treatment with Human Bone Marrow-Derived Stromal Cells Evaluated in Rats with Spinal Cord Injury. Int J Mol Sci 25(3). doi: 10.3390/ijms25031548.

Bakshi, A., Barshinger, A.L., Swanger, S.A., Madhavani, V., Shumsky, J.S., Neuhuber, B., et al. (2006). Lumbar puncture delivery of bone marrow stromal cells in spinal cord contusion: a novel method for minimally invasive cell transplantation. J Neurotrauma 23(1), 55-65. doi: 10.1089/neu.2006.23.55.

Bonilla, C., Zurita, M., Aguayo, C., Rodriguez, A., and Vaquero, J. (2014). Is the subarachnoid administration of mesenchymal stromal cells a useful strategy to treat chronic brain damage? Cytotherapy 16(11), 1501-1510. doi: 10.1016/j.jcyt.2014.07.007.

Cao, T.T., Chen, H., Pang, M., Xu, S.S., Wen, H.Q., Liu, B., et al. (2022). Dose optimization of intrathecal administration of human umbilical cord mesenchymal stem cells for the treatment of subacute incomplete spinal cord injury. Neural Regen Res 17(8), 1785-1794. doi: 10.4103/1673-5374.332151.

Cizkova, D., Novotna, I., Slovinska, L., Vanicky, I., Jergova, S., Rosocha, J., et al. (2011). Repetitive intrathecal catheter delivery of bone marrow mesenchymal stromal cells improves functional recovery in a rat model of contusive spinal cord injury. J Neurotrauma 28(9), 1951-1961. doi: 10.1089/neu.2010.1413.

Huang, Z., Jiang, J., Peng, Q., Jin, M., Dong, Y., Li, X., et al. (2026). Preclinical safety and ef fi cacy evaluation of the intrathecal transplantation of GMP-grade human umbilical cord mesenchymal stem cells for ischemic stroke. Neural Regen Res 21(3), 1172-1182. doi: 10.4103/NRR.NRR-D-24-00683.

Krupa, P., Vackova, I., Ruzicka, J., Zaviskova, K., Dubisova, J., Koci, Z., et al. (2018). The Effect of Human Mesenchymal Stem Cells Derived from Wharton's Jelly in Spinal Cord Injury Treatment Is Dose-Dependent and Can Be Facilitated by Repeated Application. Int J Mol Sci 19(5). doi: 10.3390/ijms19051503.

Lim, J.Y., Jeong, C.H., Jun, J.A., Kim, S.M., Ryu, C.H., Hou, Y., et al. (2011). Therapeutic effects of human umbilical cord blood-derived mesenchymal stem cells after intrathecal administration by lumbar puncture in a rat model of cerebral ischemia. Stem Cell Res Ther 2(5), 38. doi: 10.1186/scrt79.

Liu, G., Zhao, Z., Wang, H., Hao, C., Wang, W., Zhang, C., et al. (2022). Therapeutic Efficacy of Human Mesenchymal Stem Cells With Different Delivery Route and Dosages in Rat Models of Spinal Cord Injury. Cell Transplant 31, 9636897221139734. doi: 10.1177/09636897221139734.

Mohamadi, Y., Noori Moghahi, S.M.H., Mousavi, M., Borhani-Haghighi, M., Abolhassani, F., Kashani, I.R., et al. (2019). Intrathecal transplantation of Wharton's jelly mesenchymal stem cells suppresses the NLRP1 inflammasome in the rat model of spinal cord injury. J Chem Neuroanat 97, 1-8. doi: 10.1016/j.jchemneu.2019.01.011.

Pal, R., Gopinath, C., Rao, N.M., Banerjee, P., Krishnamoorthy, V., Venkataramana, N.K., et al. (2010). Functional recovery after transplantation of bone marrow-derived human mesenchymal stromal cells in a rat model of spinal cord injury. Cytotherapy 12(6), 792-806. doi: 10.3109/14653249.2010.487899.

Paul, C., Samdani, A.F., Betz, R.R., Fischer, I., and Neuhuber, B. (2009). Grafting of human bone marrow stromal cells into spinal cord injury: a comparison of delivery methods. Spine (Phila Pa 1976) 34(4), 328-334. doi: 10.1097/BRS.0b013e31819403ce.

Romero-Ramirez, L., Wu, S., de Munter, J., Wolters, E.C., Kramer, B.W., and Mey, J. (2020). Treatment of rats with spinal cord injury using human bone marrow-derived stromal cells prepared by negative selection. J Biomed Sci 27(1), 35. doi: 10.1186/s12929-020-00629-y.

Ruzicka, J., Machova-Urdzikova, L., Gillick, J., Amemori, T., Romanyuk, N., Karova, K., et al. (2017). A Comparative Study of Three Different Types of Stem Cells for Treatment of Rat Spinal Cord Injury. Cell Transplant 26(4), 585-603. doi: 10.3727/096368916X693671.

Satake, K., Lou, J., and Lenke, L.G. (2004). Migration of mesenchymal stem cells through cerebrospinal fluid into injured spinal cord tissue. Spine (Phila Pa 1976) 29(18), 1971-1979. doi: 10.1097/01.brs.0000138273.02820.0a.

Sun, L., Wang, F., Chen, H., Liu, D., Qu, T., Li, X., et al. (2019). Co-Transplantation of Human Umbilical Cord Mesenchymal Stem Cells and Human Neural Stem Cells Improves the Outcome in Rats with Spinal Cord Injury. Cell Transplant 28(7), 893-906. doi: 10.1177/0963689719844525.

Urdzikova, L.M., Ruzicka, J., LaBagnara, M., Karova, K., Kubinova, S., Jirakova, K., et al. (2014). Human mesenchymal stem cells modulate inflammatory cytokines after spinal cord injury in rat. Int J Mol Sci 15(7), 11275-11293. doi: 10.3390/ijms150711275.

Vanecek, V., Zablotskii, V., Forostyak, S., Ruzicka, J., Herynek, V., Babic, M., et al. (2012). Highly efficient magnetic targeting of mesenchymal stem cells in spinal cord injury. Int J Nanomedicine 7, 3719-3730. doi: 10.2147/IJN.S32824.

Vives, J., Hernandez, J., Mirabel, C., Puigdomenech-Poch, M., Romeo-Guitart, D., Marmolejo-Martinez-Artesero, S., et al. (2022). Preclinical Development of a Therapy for Chronic Traumatic Spinal Cord Injury in Rats Using Human Wharton's Jelly Mesenchymal Stromal Cells: Proof of Concept and Regulatory Compliance. Cells 11(14). doi: 10.3390/cells11142153.

Wang, L.J., Zhang, R.P., and Li, J.D. (2014). Transplantation of neurotrophin-3-expressing bone mesenchymal stem cells improves recovery in a rat model of spinal cord injury. Acta Neurochir (Wien) 156(7), 1409-1418. doi: 10.1007/s00701-014-2089-6.

Xu, Y., Peng, T.T., Huang, S., Guo, X., Luo, J., Peng, T., et al. (2025). Repeated Intrathecal Stem Cells Optimize Neuroplasticity and Motor Function in Moderate-to-Severe Cerebral Palsy of Rats. Stem Cells Int 2025, 4337435. doi: 10.1155/sci/4337435.

Yang, Y., Cao, T.T., Tian, Z.M., Gao, H., Wen, H.Q., Pang, M., et al. (2020). Subarachnoid transplantation of human umbilical cord mesenchymal stem cell in rodent model with subacute incomplete spinal cord injury: Preclinical safety and efficacy study. Exp Cell Res 395(2), 112184. doi: 10.1016/j.yexcr.2020.112184.

Zhang, R.P., Xu, C., Liu, Y., Li, J.D., and Xie, J. (2015). Visual bone marrow mesenchymal stem cell transplantation in the repair of spinal cord injury. Neural Regen Res 10(3), 404-411. doi: 10.4103/1673-5374.153688.
