# Supplementary material for: Intrathecal injection of 3D-mesenchymal stem cells attenuates disseminated neuroinflammation and improves cognitive function in controlled cortical impact rats
Source: Front Neurosci. 2026 Jul 28;20:1895538. doi: 10.3389/fnins.2026.1895538 (PMC13457469; doi:10.3389/fnins.2026.1895538)
Supplement: Supplementary file 3 [file Supplementary_file_1.docx]

**Supplementary Text 1**

**1. Supplementary Methods**

For MRI analysis, within-group lesion volume changes between day 2 and day 7 were analyzed using paired t-tests after confirmation of normality of paired differences. To further assess lesion evolution, lesion volume reduction rate was calculated as (D2 − D7) / D2 × 100% and compared between groups using an unpaired t-test. Data are presented as mean ± SD.

**2. Supplementary Results**

The paired differences in lesion volume passed the Shapiro-Wilk normality test in both the CCI+ACSF group (p = 0.878) and the CCI+3D-MSCs group (p = 0.727). Paired t-test showed no significant difference between day 2 and day 7 in the CCI+ACSF group (mean difference = 19.16 ± 21.01 mm³, t = 2.039, df = 4, p = 0.111), whereas lesion volume was significantly reduced at day 7 in the CCI+3D-MSCs group (mean difference = 34.53 ± 6.24 mm³, t = 12.367, df = 4, p < 0.001).

The lesion volume reduction rate was 18.02 ± 18.32% in the CCI+ACSF group and 38.12 ± 6.14% in the CCI+3D-MSCs group. Shapiro-Wilk testing confirmed normality in both groups, and Levene’s test indicated homogeneity of variance (F = 2.247, p = 0.172). Unpaired t-test showed that the reduction rate was significantly greater in the CCI+3D-MSCs group than in the CCI+ACSF group (t = -2.326, df = 8, p = 0.048).
